# Associations between body mass index and mortality or cardiovascular events in a general Korean population

**DOI:** 10.1371/journal.pone.0185024

**Published:** 2017-09-15

**Authors:** Kyoung Ae Kong, Junbeom Park, So-hyeon Hong, Young Sun Hong, Yeon-Ah Sung, Hyejin Lee

**Affiliations:** 1 Department of Preventive Medicine, Ewha Womans University, College of Medicine, Seoul, Korea; 2 Division of Cardiology, Department of Internal Medicine, Ewha Womans University, College of Medicine, Seoul, Korea; 3 Division of Endocrinology and Metabolism, Department of Internal Medicine, Ewha Womans University, College of Medicine, Seoul, Korea; Shanghai Institute of Hypertension, CHINA

## Abstract

**Background/Objectives:**

The relationship between body mass index (BMI) and mortality remains controversial. Furthermore, the association between BMI and cardiovascular events (CVE) is not conclusive and may differ by ethnicity. We aimed to estimate the associations between the BMI and mortality or cardiovascular disease in a general Korean population.

**Subjects/Methods:**

This study was based on a sample cohort database released by the Korean National Health Insurance Service. We analyzed a total of 415,796 adults older than 30 years of age who had undergone a national health examination at least once from 2002 to 2012. Hazard ratios for death and cardiovascular events were calculated using Cox proportional hazards models.

**Results:**

For both men and women, BMI and overall mortality showed a U-shaped association, with the lowest mortality rate among those with a BMI of 25–27.4 kg/m^2^. Compared with them, subjects with a BMI ≥ 30kg/m^2^, men with a BMI < 25 kg/m^2^, and women with a BMI < 22.5 kg/m^2^ showed significantly higher overall mortality. Additionally, men with a BMI < 22.5 kg/m^2^ and women with a BMI < 20 kg/m^2^ displayed an increased risk of cardiovascular mortality. Unlike the mortality trend, the CVD events trend showed a linearly positive association. The risk of a CVE was the lowest in men with a BMI ranging from 20 to 22.4 kg/m^2^ and in women with a BMI < 20 kg/m^2^.

**Conclusions:**

The BMI showed a U-shaped association with overall mortality, where slightly obese subjects showed the lowest rate of mortality. The CVE exhibited a linear association with the BMI, where the lowest risk was observed for normal weight subjects in a general Korean population.

## Introduction

The prevalence of individuals who are overweight or obese has increased worldwide. Obesity is closely associated with metabolic diseases, including type 2 diabetes mellitus (DM), hypertension (HTN), and cardiovascular diseases (CVD), which are the leading causes of death [1]. Although a high body mass index (BMI) is generally accepted as an indicator of obesity, the relationships between BMI and overall mortality and between BMI and cardiovascular mortality remain controversial. In contrast to conventional beliefs that obesity and overweight status are associated with increased mortality, a previous study showed that overweight status could decrease all-cause mortality [2].

The association between BMI and mortality could depend on ethnicity. In studies of East Asian populations, a U-shaped relationship between BMI and mortality was reported, and individuals with a BMI ranging from 18.5 to 27.5 kg/m^2^ displayed the lowest risk of overall mortality [3–6]. In a large meta-analysis study, mild obesity was not associated with higher mortality rates, and overweight status was associated with a significantly lower risk of all-cause mortality [7]. In Korea, subjects who were overweight or mildly obese showed the lowest risk of all-cause mortality [3, 8]. When considering the relationship between BMI and mortality, the reverse causality could be a significant limitation. So to reduce the reverse causality, previous studies suggested the possible solution such as the exclusion of smokers, subjects who already have some chronic disease, and participants dying within five years of recruitment. [9]

Cardiovascular disease is a leading cause of death, and HTN, type 2 DM, dyslipidemia, smoking, and obesity are considered cardiovascular risk factors. However, the relationships between the BMI and cardiovascular mortality and between the BMI and cardiovascular events are not entirely understood. In a meta-analysis of patients with chronic heart failure, the risks of a cardiovascular event and cardiovascular mortality were the highest in subjects with a low BMI and those who were overweight [10]. In contrast, in a study analyzing a Dutch population, obesity and overweight status had a strong impact on the development of fatal and nonfatal cardiovascular disease [11].

We analyzed a sample cohort database based on Korean National Health Insurance data to investigate the relationships between the BMI and all-cause or cardiovascular mortality, and between the BMI and cardiovascular events.

## Subjects and methods

### Data source

We used the National Sample Cohort (NSC) database released from the Korean National Health Insurance Service (NHIS; NHIS-2017-2-385), which is available to researchers through a simple application process and ethics approval. The NHIS constructed this cohort of 1,025,340 subjects (approximately 2.2% of the general population) from all beneficiaries of the Korean National Health Insurance Program and the Medical Aid Program in 2002 [12]. The cohort members were selected by systematic sampling within each of the 1,476 strata based on age, gender, eligibility status, and income level, and the sample size was proportionate to the population size of the stratum. This cohort also included an annual sample of neonates. The cohort selected is a representative sample of the Korean population, as the Korean National Health Insurance Program is a universal, mandatory health insurance program in Korea. The NSC database has gathered data for approximately one million people every year over the last 12 years.

The NSC database consists of several datasets: a dataset that contains socio-demographic information (age, sex, income, residence, and the national disability rating) of the beneficiaries from the National Health Insurance and the National Medical Aid Programs; a dataset that contains medical claims, including information on the diagnosis (recorded according to the tenth revision of the International Classification of Disease (ICD-10) codes), admission, and treatment (such as operations, procedures, prescribed medication, cost, and information on the medical service center); and a dataset that contains the cohort members of the National Health Screening Program. The National Health Screening Program in Korea provides biennial (or annual for pre-specified manual occupations) health examinations for all medical insurance subscribers (at any age), other beneficiaries aged ≥40 years (usually family members of the subscriber), and those between the ages of 44 and 66 years. This program includes a regular blood test, a chest X-ray examination, a physical examination, and a questionnaire on medical history. Approximately 70% of those eligible for a national health examination utilized these additional features. Data from the national death registration database of the Korea National Statistical Office (i.e., the date and the cause of death according to the ICD-10 codes) were linked with the NHIS NSC database.

This cohort was followed up until the study ended (the year that the data were finally released or used) or until the member was disqualified from the NHIS due to death or emigration. The July 2014 initial release of the NSC database included datasets from 2002 to 2010, and the eligibility and socio-demographic information, the medical claims dataset, the National Health Screening dataset, and the national death registration database were added and updated annually. Researchers can use the NSC data with an Institutional Review Board (IRB) approval for the study proposal and a charge for the DB through the application procedure of the NHIS. In this study, we used the 2002–2013 NHIS NSC database, and the cohort was followed until 31 December 2013.

The NHIS did not obtain informed consent individually because these data were not collected for study. The patient records of the NSC were anonymous and de-identified before the data were released by the NHIS. This study was approved by the IRB of Ewha Womans University Mokdong Hospital (IRB number: EUMC 2017-04-004).

### Study population

We analyzed adults from the cohort members of the 2002–2013 NSC dataset who were more than 30 years old (842,510 subjects), and who had taken the National Health examination at least once (505,375 subjects) between 2002 and 2012. The follow-up period began with their first health examination, and the information from this examination (e.g., the BMI and other covariates, including heath behaviors) was used as the baseline of each subject. Among them, 86,830 were excluded due to a medical history of stroke, ischemic heart disease (IHD), cancer, or chronic obstructive pulmonary disease; this information was obtained from the self-reported patient questionnaire administered during the first health examination and from the medical claim data (ICD codes I60-I69, I20-I25, C00-C97, J44) between 2002 and the year of the health examination. We also excluded subjects who lacked measurements for height, weight, blood pressure and fasting glucose level (875) as well as those who died during the year of the health examination (1,874). In total, 415,796 subjects were included in our analysis.

### Outcome variables, BMI, and covariates

The BMI was calculated as body weight in kilograms divided by height in meters squared; these values were measured during the health examinations. The BMI was divided into six categories (<20, 20−22.4, 22.5−24.9, 25−27.4, 27.5−29.9, and ≥30 kg/m^2^). Age was categorized into 10-year groups. Health behaviors, such as physical activity level, alcohol consumption, and smoking status, were explored in the questionnaire. Current smoking status was classified into smoking or not (non/ex-smoker). Alcohol consumption status was classified as heavy drinking (>2 drinks/day in males and >1 drinks/day in females) or non-heavy drinking (≤2 drinks/day in males and ≤1 in females); average daily alcohol consumption was based on data regarding the frequency of alcohol consumption and the average amount of alcohol consumed during each drinking occasion. Physical activity level was grouped into none, 1−2, and ≥3 times/week. In 2009, the assessment of physical activity changed from one question on the weekly frequency of exercise to three questions on the number of days per week of physical activity according to intensity (i.e., vigorous activity, moderate activity, and other activities such as walking); the total number of days across all three intensities was considered as the frequency of physical activity. Missing values were assigned to a separate category for each health behavior. Income was divided into quintiles, and the lowest income group included the beneficiaries of the National Medical Aid program. The family history of cardiovascular disease was based on the self-reported questionnaire responses for family history of stroke and heart disease.

Comorbid conditions of HTN and DM were identified based on the questionnaire responses at the first health examination, the medical claim data (ICD code I10-I15 or E10-E14) between 2002 and the year of the health examination, and the health examination measurements (fasting glucose ≥126 mg/dl, systolic blood pressure ≥140 mmHg or diastolic blood pressure ≥90 mmHg). Total cholesterol was classified into three groups (<200, 200−239, and ≥240 mg/dl).

The outcomes of interest were time-to-death, time-to-CVD death, and time-to-CVD incidence. We defined CVD incidence as the first admission with a diagnosis of CVD or CVD death. CVD was identified as ICD-10 codes I21-I25 and I60-I69 for the cause of death or a diagnosis reported in the medical claim data and was categorized into the following groups: IHD (ICD codes I20–I25), ischemic stroke (ICD codes I63), and hemorrhagic stroke (ICD codes I60-I62).

### Statistical analysis

The characteristics of the subjects are presented as the frequency and proportion for categorical variables. We first examined the associations between the continuous BMI and the all-cause and CVD mortality using age-adjusted Cox proportional hazards models, where the BMI was restricted to a three-knot cubic spline (using the lowest Bayesian information criterion (BIC)), and by reporting the hazard ratios (HRs). After manual inspection of the plots, we set a BMI of 25 kg/m^2^ as the reference for the all-cause mortality HRs and a BMI 21 kg/m^2^ as the reference for the CVD mortality HR. Then, we examined the same associations with a categorical BMI using Cox proportional hazards models, adjusting for age, smoking, and alcohol consumption status, levels of physical activity and income, and a family history of CVD. As a reference, we used the 25−27.4 kg/m^2^ BMI group for the mortality analysis and the 22.5−24.9 kg/m^2^ BMI group for the incidence analysis.

We also assessed the associations between BMI and all-cause and CVD mortality according to age, smoking, HTN, and DM status, and we examined the interactions between the BMI and HTN and between the BMI and DM status. In addition, we conducted a sensitivity analysis to avoid the possibility of reverse causality, in which we excluded subjects who died within less than 3 years after the baseline measurements (for analysis of the overall and CVD mortality) and additionally excluded subjects who were diagnosed with the corresponding diseases (for analysis of the CVD incidence). For overall mortality, we also conducted a sensitivity analysis excluding subjects who died within less than five years after the baseline examination. In all sensitivity analysis, we used the variable of smoking status classified into never smoker, ex-smoker, and current smoker.

If two BMI groups in the study were compared with a total number of subjects of fifty thousand and the ratio of the number of the subject of 4, and the proportions of subjects having the events in the control group is 2% (for overall mortality) or 5% (for CVD events) during study period, the two-sided tests of whether the hazard ratio is one achieve 81% or 99% power at a 0.05 significance level when the hazard ratio is actually 1.25.

All analyses were conducted separately for each sex. The *P* values were generated using two-tailed tests, and *P* values <0.05 were considered statistically significant. All statistical analyses were performed using SAS (version 9.4, SAS Institute, Cary, NC), STATA (version 10.0, StataCorp, College Station, Texas), and Prism (Version 5.03, GraphPad Software Inc., La Jolla, CA) software.

## Results

A total of 415,796 subjects were included in our analysis, and the median follow-up duration was 7.0 years (interquartile range: 4–11 years). During the study period, 11,058 subjects (7,257 men and 3,801 women) died, among whom 1,482 subjects (882 men and 600 women) died from CVD. The subject demographics are shown in Table 1. At enrollment, the mean BMI was 24.1 ± 3.1 kg/m^2^ for men and 23.2 ± 3.3 kg/m^2^ for women. The subjects aged <50 years and ≥60 years comprised 71% and 12% of all men, respectively, and 63% and 17% of all women, respectively. A total of 48.1% of men and 3.8% of women were current smokers, and 6.4% of men and 7.1% of women had a family history of CVD.

**Table 1:** Demographic and clinical characteristics of the subjects.

	Male (n = 218,388)	Female (n = 197,408)
Age (years)		
- 20–39	92,645 (42.4%)	45,232 (22.9%)
- 40–49	62,200 (28.5%)	78,318 (39.7%)
- 50–59	36,732 (16.8%)	40,564 (20.5%)
- 60–69	20,026 (9.2%)	22,917 (11.6%)
- >70	6,785 (3.1)	10,377 (5.3%)
BMI (kg/m^2^)	24.1 ± 3.1	23.2 ± 3.3
Smoking status		
- Current smoker	104,955 (48.1%)	7,575 (3.8%)
- Ex-smoker	21,047 (9.6%)	2,176 (1.1%)
- Never smoker	71,278 (32.6%)	180,390 (91.38%)
Alcohol consumption status		
- Heavy drinker	52,545 (24.1%)	11,396 (5.8%)
- Non-heavy drinker	161,868 (74.1%)	179,494 (90.9%)
Physical Activity		
- None	105,306 (48.2%)	120,707 (61.1%)
- 1–2 times/week	64,019 (29.3%)	35,688 (18.0%)
- ≥ 3 times/week	44,480 (20.4%)	37,104 (18.7%)
Family history of CVD	13,989 (6.4%)	14,003 (7.1%)
Systolic BP (mmHg)	126 ± 15	120 ± 17
Diastolic BP (mmHg)	79 ± 10	75 ± 11
Fasting blood glucose (mg/dl)	98 ±30	94 ± 24
Total cholesterol (mg/dl)	196 ± 37	195 ± 37

BMI, body mass index; CVD, cardiovascular disease; BP, blood pressure

**Fig 1:**
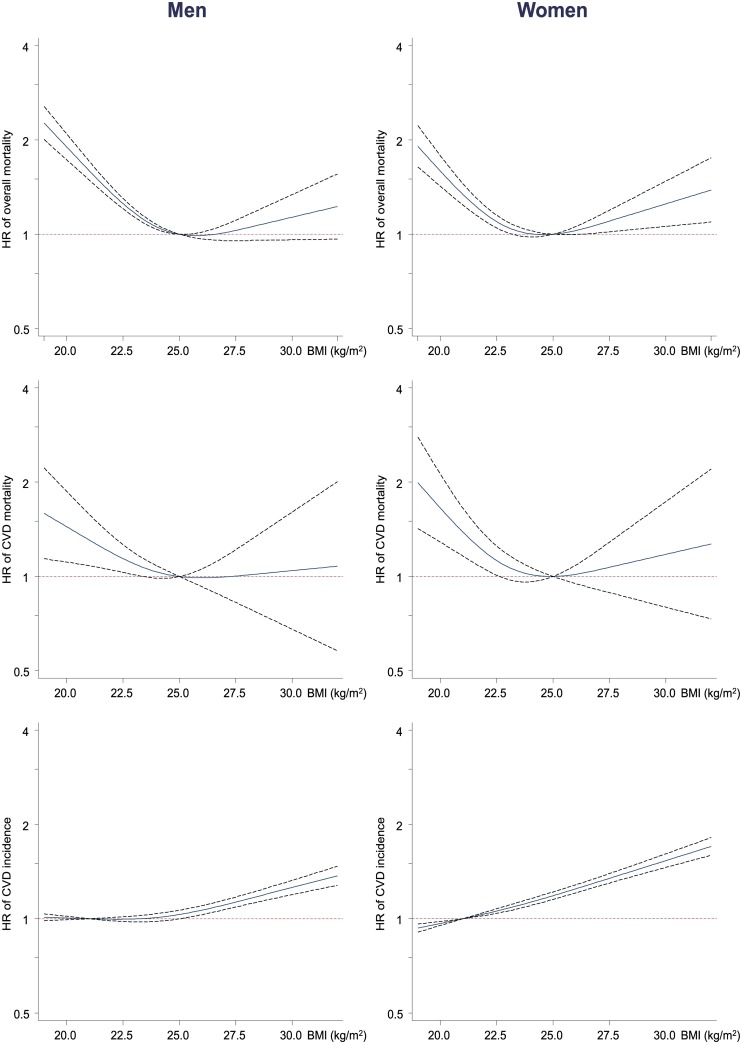
Association between continuous BMI and overall mortality, CVD mortality, and CVD events as a cubic spline using Cox regression models adjusted only for age. For overall and CVD mortality rates, the reference BMI was set at 25 kg/m^2^; for CVD events, the reference BMI was set at 21 kg/m^2^. BMI, body mass index; CVD, cardiovascular disease; HR, hazard ratio.

Fig 1 shows the non-linear associations between the continuous BMI and overall mortality, CVD mortality, and CVD events. The associations between the BMI and age-adjusted overall and CVD mortality showed a U-shaped pattern, with the lowest HR, observed in subjects with a BMI ranging from approximately 24 to 26 kg/m^2^. The risk of a CVD event increased nearly linearly across the entire range of BMI values in women and BMI values greater than 22.5 kg/m^2^ in men.

The association between a categorical BMI and overall mortality is shown in S1 Table. For both men and women, we observed a U-shaped association, with the lowest mortality rate in the 25–27.4 kg/m^2^ BMI group. The highest mortality rate was observed in the < 20 kg/m^2^ BMI group (HR: men 1.91, women 1.50), and this relationship was more prominent in men (S1 Table). Male subjects with a BMI < 25 kg/m^2^ or a BMI ≥ 30 kg/m^2^ and female subjects with a BMI < 22.5 kg/m^2^ or a BMI ≥ 30 kg/m^2^ showed significantly higher overall mortality than male or female subjects with a BMI of 25–27.4 kg/m^2^. The overall mortality HRs were not different according to smoking status (among men and women) and HTN status (among men). However, the effects of BMI values <20.0 kg/m^2^ and 20.0–22.4 kg/m^2^ on overall mortality were significantly stronger among men with DM than among men without DM (*P*_interaction_ = 0.020). Among women with HTN and DM, those with a BMI ranging from 27.5 to 29.9 kg/m^2^ showed HRs that were not different those with a BMI ranging from 25 to 27.4 kg/m^2^, whereas among women without HTN and DM, those with a BMI ranging from 27.5 to 29.9 kg/m^2^ displayed a higher risk of overall mortality than those with a BMI ranging from 25 to 27.4 kg/m^2^ (*P*_interaction_ = 0.015 for HTN and <0.001 for DM) (S1 Fig and S1 Table). These patterns of the association between BMI and overall mortality were maintained in subgroup analyses excluding subjects who died within less than 3 or 5 years after baseline examination (S2 Table).

We also investigated the relationship between CVD mortality and BMI (S3 Table). For men and women, we observed a U-shaped association, with the lowest mortality rate in the 25–27.4 kg/m^2^ BMI group in men and 27.5–29.9 kg/m^2^ BMI group in women. Male subjects with a BMI < 22.5 kg/m^2^ and female subjects with a BMI < 20 kg/m^2^ showed significantly higher CVD mortality HRs than their counterparts with a BMI of 25–27.4 kg/m^2^; the highest CVD mortality was observed in the < 20 kg/m^2^ BMI group (HR: men 1.35, women HR 1.51). IHD and ischemic stroke mortality were not significantly different according to BMI category, and the relationships were similar to those for CVD mortality. For women, hemorrhagic stroke mortality was higher among those with a BMI < 22.5 kg/m^2^ than among those with a BMI from 25–27.4 kg/m^2^. In subgroup analyses excluding subjects who died within less than 3 years after baseline examination, the patterns of the association between BMI and CVD mortality were similar, although the magnitude of the HRs reduced (and not significant) among men and increased among women (S4 Table). CVD mortality was not significantly different according to smoking status, HTN status, or DM status; however, the low number of CVD deaths observed in each stratum reduced the power of the interaction test for the analysis of these categories (S5 and S6 Tables).

**Fig 2:**
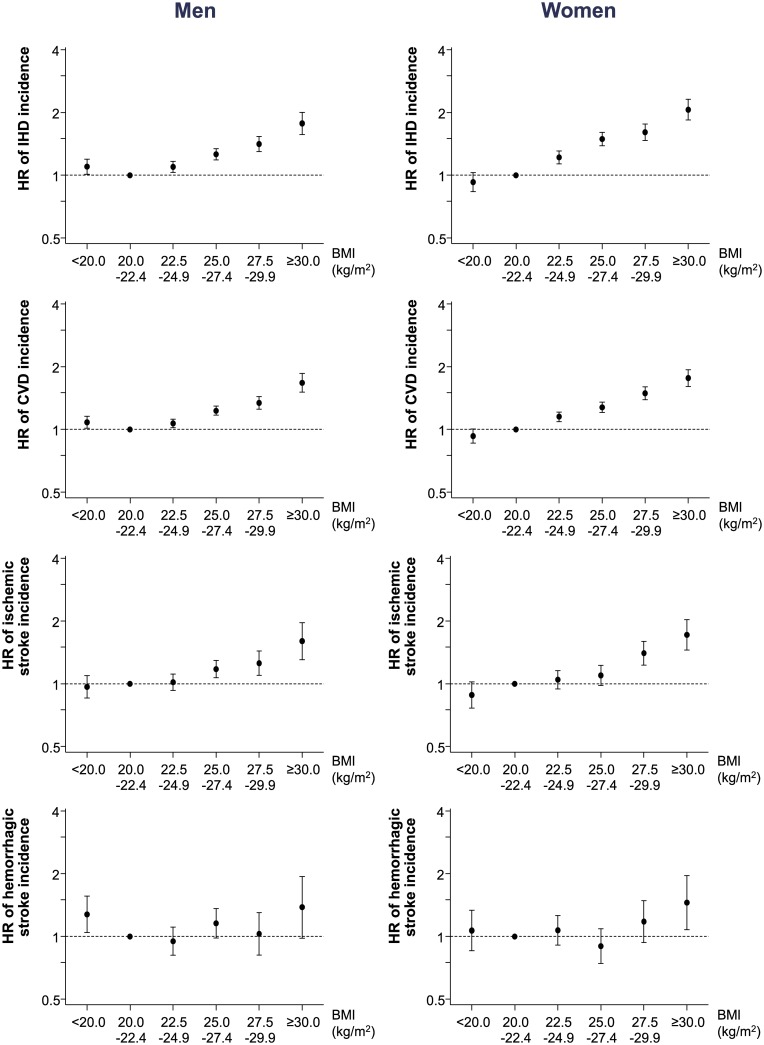
Cardiovascular disease event risk according to the categorical body mass index. HRs were adjusted for gender, health behaviors (smoking, alcohol consumption, physical activity), income, and family history of CVD. BMI, body mass index; CVD, cardiovascular disease; HR, hazard ratio.

We analyzed CVD events according to the BMI (Fig 2 and S3 Table). The CVD events trend was different from the mortality trend in both men and women. The risk of a CVD event linearly increased with BMI except for the <20 kg/m^2^ BMI group in men, which showed a slightly higher risk than the reference BMI group of 20.0–22.4 kg/m^2^ (HR 1.08, 95% CI 1.01–1.16). The associations between BMI and an IHD event and an ischemic stroke event were similar to the association between BMI and a CVD event. For hemorrhagic stroke events, the increase in risk was less evident; for men with a BMI < 20 kg/m^2^, the increase in the risk of a hemorrhagic stroke event was similar to the risk of hemorrhagic stroke mortality. These patterns of the association between BMI and CVD event were maintained in subgroup analyses excluding subjects who died within less than 3 years after baseline examination (S4 Table).

**Fig 3:**
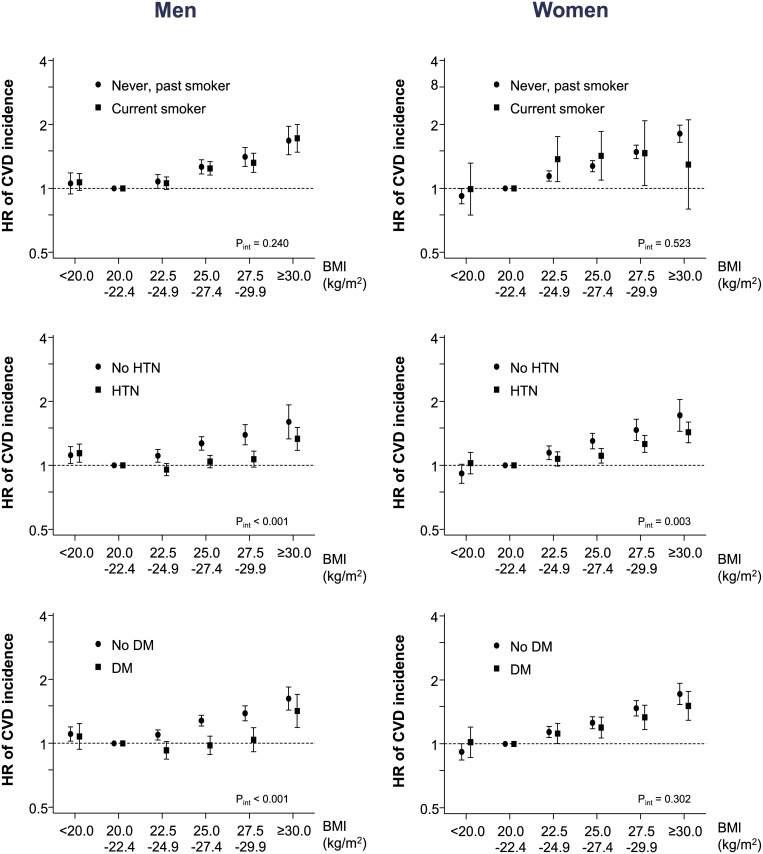
Cardiovascular disease event risk according to smoking status, hypertension, and diabetes status. HRs were adjusted for gender, health behaviors (smoking, alcohol consumption, physical activity), income, and family history of CVD. BMI, body mass index; CVD, cardiovascular disease; DM, diabetes mellitus; HR, hazard ratio; HTN, hypertension.

The CVD event HRs were not different according to smoking status. However, the effect of a higher BMI on the risk of a CVD event was significantly weaker among those with HTN and DM than among those without these diseases (*P*_interaction_<0.01 for HTN among men and women and for DM among men) (Fig 3 and S7 and S8 Tables).

## Discussion

In our study of more than 400,000 participants, we observed that BMI showed a U-shaped association with overall mortality with the lowest mortality in slightly obese subjects (BMI 25~27.4 kg/m^2^), and highest mortality in BMI values <20 kg/m^2^ in general Korean population. An increased risk of death was found in BMI <22.5 kg/m^2^ in men and BMI<20 kg/m^2^ in women for overall CVD, compared with those with a BMI of 25–27.4 kg/m^2^. On the other hand, the risk for cardiovascular events presented a linear pattern, with the lowest risk in normal weight subjects. The smoking status did not affect the relationship between BMI and mortality or CVD event, and the association was maintained in subgroup analyses excluding those who died within less than 3 or 5 years after recruitment. This suggested that the BMI was related to the overall and CVD mortality/event.

We used the NSC database from the NHIS. The Korean universal health insurance system was initiated in 1963 for all citizens, and universal healthcare coverage was achieved in 1989. We used the sample cohort of 1,025,340 participants released by the NHIS, so it represents the total Korean population [12]. Obesity and overweight status have been linked with DM, cardiovascular disease, and some cancers. Therefore, obesity and overweight status were expected to be associated with higher mortality. However, many previous epidemiologic studies have reported conflicting results. A study analyzing a large East Asian population showed that the lowest risk of death was observed among persons with a BMI ranging from 22.6 to 27.5 kg/m^2^, and that risk of death was elevated among persons with BMI values either higher or lower than that range. Moreover, those with a BMI of 15.0 kg/m^2^ or less showed the highest risk of mortality [4]. The association between BMI and mortality could be dependent on race. In a large prospective cohort of Caucasian and African-American U.S. adults, a higher BMI was most strongly associated with a higher risk of mortality, and all levels of obesity and overweight status were associated with a significantly higher risk of mortality compared with the reference BMI (22.5–24.9 kg/m^2^) [13]. In a Spanish study, BMI >35 kg/m^2^ is an important predictor of both overall mortality and the combination of cardiovascular morbidity plus all-cause mortality [14]. In another Western study, all-cause mortality increased with increasing BMI in adults without DM but decreased with increasing BMI among adults with DM [15]. Our study found a U-shaped relationship between BMI and overall mortality, and this result was similar to that of previous studies analyzing Asian groups. The lowest mortality risk was observed in the 25–27.4 kg/m^2^ BMI group; these subjects are slightly obese according to the Asian obesity criteria of the World Health Organization [16]. In a previous Korean study that examined the association between body weight and the risk of death, the risk was lowest among persons with a BMI ranging from 23.0 to 24.9 kg/m^2^ [3]. However, this study was based on data from the early 1990s, whereas our study was based on data obtained after the year 2000. We observed that the lowest-risk BMI regarding overall mortality was higher than the current range for normal BMI.

We found that CVD mortality rate showed a U-shaped association with BMI and that the lowest BMI group showed the highest CVD mortality rate. Several meta-analyses, mostly based on Western populations, evaluated the association between BMI and CVD mortality [2, 17, 18]. In a prospective study collaboration, which included mostly cohorts from Western countries, the BMI positively correlated with coronary heart disease mortality for BMI values ranging from 20 to 40 kg/m^2^, with the lowest risk observed among those with a BMI ranging from 20 to 22.5 kg/m^2^ [19]). Moreover, in a study of East Asian subjects, a U-shaped association was observed between the BMI and the risk of CVD death (6). In the cohort analyzed in our study, the association between BMI and CVD mortality differed according to the CVD subtype. Ischemic heart disease and ischemic stroke showed a U-shaped association, whereas hemorrhagic stroke showed a U-shaped association only in women and a reverse J-shaped association in men. Additionally, the lowest-risk mortality group was slightly different according to the different CVD subtypes. Men with a BMI ranging from 25.0 to 27.4 kg/m^2^ displayed the lowest CVD mortality rate, whereas women with a BMI ranging from 27.5 to 29.9 kg/m^2^ displayed the lowest mortality rate. A low BMI may indicate a low level of circulating total cholesterol or triglycerides, which is associated with an increased risk of hemorrhagic stroke according to previous studies [20–22]; our study results are consistent with these findings.

CVD is a common cause of death worldwide, and the rate of CVD deaths will likely continue to increase [23]. Obesity and overweight status are cardiovascular disease risk factors according to studies conducted in Europe and North America. Additionally, in an East Asian meta-analysis study, the authors reported continuous, positive associations between the BMI and the risks of ischemic stroke, hemorrhagic stroke, and ischemic heart disease [24]. We estimated the risk of a CVD event among different BMI groups, and we observed that cardiovascular events showed a linear pattern, with the lowest risk among normal weight subjects. The incidences of ischemic heart disease and ischemic stroke increased with increasing BMI; however, for hemorrhagic stroke, only subjects with a BMI ≥ 30 kg/m^2^ and men with a BMI < 20 kg/m^2^ showed increased risk. This result is consistent with those of previous studies that showed that the BMI is related to ischemic stroke risk [25, 26]. The association between BMI and hemorrhagic stroke is controversial. Most Western studies have shown no association [27, 28] or an inverse association [29] between BMI and hemorrhagic stroke; however, studies in Asian populations have reported a J- or U-shaped association between the BMI and hemorrhagic stroke risk [26, 30, 31].

Interestingly, the associations between BMI and mortality and cardiovascular events show different patterns. The BMI showed a U-shaped association with overall mortality and cardiovascular mortality, but we observed that BMI was positively associated with the risk of CVD throughout the range. This linear pattern was consistent with the studies using nonfatal CVD or combined fatal and nonfatal CVD as an outcome [11, 32–34]. The difference between mortality and incidence existed in low BMI—those with a low BMI were at low risk of CVD event but at high risk of overall/CVD death. It was unlikely to be caused by reverse causation, because we excluded subjects with existing chronic diseases such as cancers and chronic obstructive pulmonary disease in the analyses, and conducted sensitivity analyses with exclusion of deaths within <3 or 5 years of follow-up and by smoking status. One of the possible reasons is case fatality. Among those who experienced a CVD event during the follow-up period in our study, the proportions who died from CVD or any cause were 10.5% and 30.8% in the group with a BMI of <20 kg/m^2^, and 4.3% and 11.6% with a BMI of ≥30 kg/m^2^. Those with a low BMI were at low risk of CVD, but at high risk of dying once, they experience a CVD event. Those with a high BMI were at higher risk of a CVD event, but at lower risk of dying should they experience one. These results were similar to the phenomenon called the obesity paradox, in which patients with a higher BMI have better survival compared to those with a lower BMI [35–37]. Better nutritional status and metabolic reserves more resistant to the catabolic disease status, and more improved immune response in obese patients is mentioned as a part of the pathophysiology. Furthermore, BMI has inherent inadequacy to represent the body fat distribution or body composition and to differentiate lean and fat mass [35–39]. A low BMI could indicate a small thigh circumference or a low total fat-free mass, which are risk factors for total mortality and cardiovascular mortality [40–42]. In addition, obese individuals usually receive more aggressive medical treatment including statins or anti-platelet agents, which could contribute to a lower mortality rate. However, the existence and the mechanism of obesity paradox itself is still unclear. Moreover, it has been reported in the studies mainly of the elderly population and the patients with heart failure, coronary heart disease, diabetes mellitus, cancer, kidney disease, chronic obstructive lung disease, or community-acquired pneumonia, not of the general population.

Also of note is that subjects with DM displayed an inverse correlation between mortality rate and BMI, whereby lower BMI values were associated with higher mortality rates. This finding indicates that DM modifies the BMI-mortality relationship and may be associated with different effects between the BMI and mortality rate than those observed for the general population.

Our study has several strengths. First, the NHIS database contains representative samples of population-based data, which are large-scale and stable. We were able to obtain nationwide health insurance data, including questionnaires, disease states, and laboratory and anthropometric data, that was generated by a public institution. Second, low body weight caused by illness could distort the association between underweight status and health due to reverse causation. However, to reduce this type of bias, we excluded subjects with a history of stroke, ischemic heart disease, cancers, and chronic obstructive pulmonary disease, and we analyzed the mortality data using several models that adjusted for multiple CVD risk factors. Additionally, the sensitivity analysis, which excluded subjects diagnosed with CVD or cancer as well as those who died during the first 3 years of follow-up, showed similar patterns.

However, the NHIS database also has limitations. Although the cohort consisted of more than one million participants with the national representativeness, the number of deaths and the resultant power of the study might not have been enough to explore the association between the cause-specific mortality and each BMI category, particularly in subgroup analyses according to smoking or chronic disease status. The disease codes listed may not reflect the real disease status of the subjects because the codes are used for insurance claim purposes. This is an inherent limitation of the use of insurance databases. Additionally, there could be self-referral and selection bias, as the subjects who are more interested in their health are more likely to participate in routine health examinations, although this bias might not be large because NHIS health examinations are mandatory in Korea. A lack of evaluation of some main CVD risk factors such as LDL-cholesterol, HDL-cholesterol, serum uric acid and glomerular filtration rate in the national health examination was also another limitation, but it might not have much biased the association between BMI and CVD mortality/incidence because most of these metabolic and biologic factors were considered as mediating factors rather than as confounding factors. Moreover, we used the BMI as a marker of obesity; however, the BMI may not represent a subject’s obesity status accurately because it cannot distinguish between relative muscle and fat composition.

## Conclusions

In this study, we observed increased overall mortality risk among subjects with a BMI greater than 30.0 kg/m^2^ or lower than 22.5 kg/m^2^ in a general Korean population. Additionally, we found an increased risk of CVD among subjects in the overweight and obese BMI groups. Therefore, health care professionals should provide personalized treatment plans to help subjects maintain an optimal BMI. Moreover, further research on body composition in relation to health outcomes is needed.

## Supporting information

S1 Table Multivariate hazard ratios for overall mortality according to body mass index. All HRs were adjusted for age, behavior, income, and family history of cardiovascular disease. Ex-smoker group among women was not presented due to the small number. BMI, body mass index; HTN, hypertension; DM, diabetes mellitus; HR, hazard ratio.
(DOCX)

S2 Table Multivariate hazard ratios for overall mortality according to body mass index, excluding subjects who died within less than 3 or 5 years after the baseline examination. All HRs were adjusted for age, behavior, income, and family history of cardiovascular disease. Ex-smoker group among women was not presented due to the small number. BMI, body mass index; HTN, hypertension; DM, diabetes mellitus; HR, hazard ratio.
(DOCX)

S3 Table Multivariate hazard ratios for cardiovascular disease mortality and a cardiovascular disease event according to body mass index. All HRs were adjusted for age, behavior, income, and family history of cardiovascular disease. BMI, body mass index; CVD, cardiovascular disease; HR, hazard rati.
(DOCX)

S4 Table Multivariate hazard ratios for cardiovascular disease mortality and a cardiovascular disease event according to body mass index, excluding subjects who died within less than 3 years after baseline examination. All HRs were adjusted for age, behavior, income, and family history of cardiovascular disease. BMI, body mass index; CVD, cardiovascular disease; HR, hazard ratio.
(DOCX)

S5 Table Multivariate hazard ratios for cardiovascular disease mortality according to body mass index. All HRs were adjusted for age, behavior, income, and family history of cardiovascular disease. Ex-smoker group among women was not presented due to the small number. BMI, body mass index; HTN, hypertension; DM, diabetes mellitus; HR, hazard ratio.
(DOCX)

S6 Table Multivariate hazard ratios for cardiovascular disease mortality according to body mass index, excluding subjects who died within less than 3 years after baseline examination. All HRs were adjusted for age, behavior, income, and family history of cardiovascular disease. Ex-smoker group among women was not presented due to the small number. BMI, body mass index; HTN, hypertension; DM, diabetes mellitus; HR, hazard ratio.
(DOCX)

S7 Table Multivariate hazard ratios for the occurrence of a cardiovascular disease event according to body mass index. All HRs were adjusted for age, behavior, income, and family history of cardiovascular disease. Ex-smoker group among women was not presented due to the small number. BMI, body mass index; HTN, hypertension; DM, diabetes mellitus; HR, hazard ratio.
(DOCX)

S8 Table Multivariate hazard ratios for the occurrence of a cardiovascular disease event according to body mass index, excluding subjects who died or were diagnosed as the corresponding disease within less than 3 years after baseline examination. All HRs were adjusted for age, behavior, income, and family history of cardiovascular disease. Ex-smoker group among women was not presented due to the small number. BMI, body mass index; HTN, hypertension; DM, diabetes mellitus; HR, hazard ratio.
(DOCX)

S1 Fig Overall mortality risk according to smoking status, hypertension, and diabetes mellitus status. HRs were adjusted for gender, health behaviors (smoking, alcohol consumption, physical activity), income, and family history of CVD. BMI, body mass index; CVD, cardiovascular disease; DM, diabetes mellitus; HR, hazard ratio; HTN, hypertension.
(DOCX)

## References

[1] Wilson PW, D'Agostino RB, Sullivan L, Parise H, Kannel WB. Overweight and obesity as determinants of cardiovascular risk: the Framingham experience. Archives of Internal Medicine. 2002; 162: 1867–72. 12196085 10.1001/archinte.162.16.1867

[2] Feigin VL, Rinkel GJ, Lawes CM, Algra A, Bennett DA, van Gijn J, et al Risk factors for subarachnoid hemorrhage: an updated systematic review of epidemiological studies. Stroke. 2005; 36: 2773–80. doi: 10.1161/01.STR.0000190838.02954.e8 16282541 10.1161/01.STR.0000190838.02954.e8

[3] Jee SH, Sull JW, Park J, Lee SY, Ohrr H, Guallar E, et al Body-mass index and mortality in Korean men and women. The New England Journal of Medicine. 2006; 355: 779–87. doi: 10.1056/NEJMoa054017 16926276 10.1056/NEJMoa054017

[4] Zheng W, McLerran DF, Rolland B, Zhang X, Inoue M, Matsuo K, et al Association between body-mass index and risk of death in more than 1 million Asians. The New England Journal of Medicine. 2011; 364: 719–29. doi: 10.1056/NEJMoa1010679 21345101 10.1056/NEJMoa1010679 PMC4008249

[5] Gu D, He J, Duan X, Reynolds K, Wu X, Chen J, et al Body weight and mortality among men and women in China. JAMA. 2006; 295: 776–83. doi: 10.1001/jama.295.7.776 16478900 10.1001/jama.295.7.776

[6] Chen Y, Copeland WK, Vedanthan R, Grant E, Lee JE, Gu D, et al Association between body mass index and cardiovascular disease mortality in east Asians and south Asians: pooled analysis of prospective data from the Asia Cohort Consortium. BMJ. 2013; 347: f5446 doi: 10.1136/bmj.f5446 24473060 10.1136/bmj.f5446 PMC3788174

[7] Flegal KM, Kit BK, Orpana H, Graubard BI. Association of all-cause mortality with overweight and obesity using standard body mass index categories: a systematic review and meta-analysis. JAMA. 2013; 309: 71–82. doi: 10.1001/jama.2012.113905 23280227 10.1001/jama.2012.113905 PMC4855514

[8] Kim NH, Lee J, Kim TJ, Choi KM, Baik SH, Choi DS, et al Body Mass Index and Mortality in the General Population and in Subjects with Chronic Disease in Korea: A Nationwide Cohort Study (2002–2010). PLoS ONE, 2015; 10: e0139924 doi: 10.1371/journal.pone.0139924 26462235 10.1371/journal.pone.0139924 PMC4604086

[9] Global BMI Mortality Collaboration, Di Angelantonio E, Bhupathiraju ShN, Wormser D, Gao P, Kaptoge S, et al Body-mass index and all-cause mortality: individual-participant-data meta-analysis of 239 prospective studies in four continents. Lancet. 2016; 388: 776–86. doi: 10.1016/S0140-6736(16)30175-1 27423262 10.1016/S0140-6736(16)30175-1 PMC4995441

[10] Sharma A, Lavie CJ, Borer JS, Vallakati A, Goel S, Lopez-Jimenez F, et al Meta-analysis of the relation of body mass index to all-cause and cardiovascular mortality and hospitalization in patients with chronic heart failure. American Journal of Cardiology. 2015; 115: 1428–34. doi: 10.1016/j.amjcard.2015.02.024 25772740 10.1016/j.amjcard.2015.02.024

[11] van Dis I, Kromhout D, Geleijnse JM, Boer JM, Verschuren WM. Body mass index and waist circumference predict both 10-year nonfatal and fatal cardiovascular disease risk: study conducted in 20,000 Dutch men and women aged 20–65 years. European journal of cardiovascular prevention and rehabilitation. 2009; 16: 729–34. doi: 10.1097/HJR.0b013e328331dfc0 19809330 10.1097/HJR.0b013e328331dfc0

[12] Lee J, Lee JS, Park SH, Shin SA, Kim K. Cohort Profile: The National Health Insurance Service-National Sample Cohort (NHIS-NSC), South Korea. International Journal of Epidemiology. 2016; doi: 10.1093/ije/dyv319 26822938 10.1093/ije/dyv319

[13] Patel AV, Hildebrand JS, Gapstur SM. Body mass index and all-cause mortality in a large prospective cohort of white and black U.S. Adults. PLoS ONE. 2014; 9: e109153 doi: 10.1371/journal.pone.0109153 25295620 10.1371/journal.pone.0109153 PMC4189918

[14] Ponce-Garcia I, Simarro-Rueda M, Carbayo-Herencia JA, Divisón-Garrote JA, Artigao-Ródenas LM, Botella-Romero F, et al Prognostic value of obesity on both overall mortality and cardiovascular disease in the general population. PLoS One. 2015;10: e0127369 doi: 10.1371/journal.pone.0127369 25992570 10.1371/journal.pone.0127369 PMC4438865

[15] Jackson CL, Yeh HC, Szklo M, Hu FB, Wang NY, Dray-Spira R, et al Body-Mass Index and All-Cause Mortality in US Adults With and Without Diabetes. The Journal of General Internal Medicine. 2014; 29: 25–33. doi: 10.1007/s11606-013-2553-7 23929218 10.1007/s11606-013-2553-7 PMC3889975

[16] World Health Organization. Obesity: preventing and managing the global epidemic. Report of a WHO consultation. World Health Organization technical report series. 2000; 894: i–xii, 1–253. 11234459

[17] Strazzullo P, D'Elia L, Cairella G, Garbagnati F, Cappuccio FP, Scalfi L. Excess body weight and incidence of stroke: meta-analysis of prospective studies with 2 million participants. Stroke. 2010; 41: e418–26 doi: 10.1161/STROKEAHA.109.576967 20299666 10.1161/STROKEAHA.109.576967

[18] McGee DL. Body mass index and mortality: a meta-analysis based on person-level data from twenty-six observational studies. Annals of Epidemiology. 2005; 15: 87–97. doi: 10.1016/j.annepidem.2004.05.012 15652713 10.1016/j.annepidem.2004.05.012

[19] Whitlock G, Lewington S, Sherliker P, Clarke R, Emberson J, Halsey J, et al Body-mass index and cause-specific mortality in 900 000 adults: collaborative analyses of 57 prospective studies. Lancet. 2009; 373: 1083–96. doi: 10.1016/S0140-6736(09)60318-4 19299006 10.1016/S0140-6736(09)60318-4 PMC2662372

[20] Bonaventure A, Kurth T, Pico F, Barberger-Gateau P, Ritchie K, Stapf C, et al Triglycerides and risk of hemorrhagic stroke vs. ischemic vascular events: The Three-City Study. Atherosclerosis. 2010; 210: 243–8. doi: 10.1016/j.atherosclerosis.2009.10.043 19963214 10.1016/j.atherosclerosis.2009.10.043

[21] Iso H, Jacobs DR Jr, Wentworth D, Neaton JD, Cohen JD. Serum cholesterol levels and six-year mortality from stroke in 350,977 men screened for the multiple risk factor intervention trial. N Engl J Med 1989; 320: 904–10. doi: 10.1056/NEJM198904063201405 2619783 10.1056/NEJM198904063201405

[22] Sturgeon JD, Folsom AR, Longstreth WT Jr, Shahar E, Rosamond WD, Cushman M. Risk factors for intracerebral hemorrhage in a pooled prospective study. Stroke 2007; 38: 2718–25. doi: 10.1161/STROKEAHA.107.487090 17761915 10.1161/STROKEAHA.107.487090

[23] Yusuf S, Reddy S, Ounpuu S, Anand S. Global burden of cardiovascular diseases: part I: general considerations, the epidemiologic transition, risk factors, and impact of urbanization. Circulation. 2001; 104: 2746–53. 11723030 10.1161/hc4601.099487

[24] Ni Mhurchu C, Rodgers A, Pan WH, Gu DF, Woodward M. Body mass index and cardiovascular disease in the Asia-Pacific Region: an overview of 33 cohorts involving 310 000 participants. International Journal of Epidemiology. 2004; 33: 751–8. doi: 10.1093/ije/dyh163 15105409 10.1093/ije/dyh163

[25] Kurth T, Gaziano JM, Berger K, Kase CS, Rexrode KM, Cook NR, et al Body mass index and the risk of stroke in men. Archives of internal medicine. 2002; 162: 2557–62. 12456227 10.1001/archinte.162.22.2557

[26] Song YM, Sung J, Davey Smith G, Ebrahim S. Body mass index and ischemic and hemorrhagic stroke: a prospective study in Korean men. Stroke. 2004; 35: 831–6. doi: 10.1161/01.STR.0000119386.22691.1C 15001798 10.1161/01.STR.0000119386.22691.1C

[27] Njolstad I, Arnesen E, Lund-Larsen PG. Body height, cardiovascular risk factors, and risk of stroke in middle-aged men and women. A 14-year follow-up of the Finnmark Study. Circulation. 1996; 94: 2877–82. 8941116 10.1161/01.cir.94.11.2877

[28] Kurth T, Gaziano JM, Rexrode KM, Kase CS, Cook NR, Manson JE, et al Prospective study of body mass index and risk of stroke in apparently healthy women. Circulation. 2005; 111: 1992–8. doi: 10.1161/01.CIR.0000161822.83163.B6 15837954 10.1161/01.CIR.0000161822.83163.B6

[29] Rodriguez BL, D'Agostino R, Abbott RD, Kagan A, Burchfiel CM, Yano K, et al Risk of hospitalized stroke in men enrolled in the Honolulu Heart Program and the Framingham Study: A comparison of incidence and risk factor effects. Stroke. 2002; 33: 230–6. 11779915 10.1161/hs0102.101081

[30] Cui R, Iso H, Toyoshima H, Date C, Yamamoto A, Kikuchi S, et al Body mass index and mortality from cardiovascular disease among Japanese men and women: the JACC study. Stroke. 2005; 36: 1377–82. doi: 10.1161/01.STR.0000169925.57251.4e 15920029 10.1161/01.STR.0000169925.57251.4e

[31] Saito I, Iso H, Kokubo Y, Inoue M, Tsugane S. Body mass index, weight change and risk of stroke and stroke subtypes: the Japan Public Health Center-based prospective (JPHC) study. International Journal of Obesity. 2011; 35:283–91. doi: 10.1038/ijo.2010.131 20603628 10.1038/ijo.2010.131

[32] Hu G, Tuomilehto J, Silventoinen K, Barengo N, Jousilahti P. Joint effects of physical activity, body mass index, waist circumference and waist-to-hip ratio with the risk of cardiovascular disease among middle-aged Finnish men and women. Eur Heart J. 2004;25:2212–9. doi: 10.1016/j.ehj.2004.10.020 15589638 10.1016/j.ehj.2004.10.020

[33] Rexrode KM, Buring JE, Manson JE. Abdominal and total adiposity and risk of coronary heart disease in men. Int J Obes Relat Metab Disord. 2001;25:1047–56. doi: 10.1038/sj.ijo.0801615 11443505 10.1038/sj.ijo.0801615

[34] Gelber RP, Gaziano JM, Orav EJ, Manson JE, Buring JE, Kurth T. Measures of obesity and cardiovascular risk among men and women. J Am Coll Cardiol. 2008;52:605–15. doi: 10.1016/j.jacc.2008.03.066 18702962 10.1016/j.jacc.2008.03.066 PMC2671389

[35] Lavie CJ, De Schutter A, Parto P, Jahangir E, Kokkinos P, Ortega FB, et al Obesity and Prevalence of Cardiovascular Diseases and Prognosis-The Obesity Paradox Updated. Prog Cardiovasc Dis. 2016;58:537–47. doi: 10.1016/j.pcad.2016.01.008 26826295 10.1016/j.pcad.2016.01.008

[36] Braun N, Gomes F, Schütz P. "The obesity paradox" in disease—is the protective effect of obesity true? Swiss Med Wkly. 2015;145:w14265 doi: 10.4414/smw.2015.14265 26709887 10.4414/smw.2015.14265

[37] Jahangir E, De Schutter A, Lavie CJ. Low weight and overweightness in older adults: risk and clinical management. Prog Cardiovasc Dis. 2014;57:127–33. doi: 10.1016/j.pcad.2014.01.001 25216611 10.1016/j.pcad.2014.01.001

[38] Prado CM, Gonzalez MC, Heymsfield SB. Body composition phenotypes and obesity paradox. Curr Opin Clin Nutr Metab Care. 2015;18:535–51. doi: 10.1097/MCO.0000000000000216 26335310 10.1097/MCO.0000000000000216

[39] Antonopoulos AS, Oikonomou EK, Antoniades C, Tousoulis D. From the BMI paradox to the obesity paradox: the obesity-mortality association in coronary heart disease. Obes Rev. 2016;17:989–1000. doi: 10.1111/obr.12440 27405510 10.1111/obr.12440

[40] Heitmann BL, Frederiksen P. Thigh circumference and risk of heart disease and premature death: prospective cohort study. BMJ. 2009; 339: b3292 doi: 10.1136/bmj.b3292 19729416 10.1136/bmj.b3292 PMC2737606

[41] Heitmann BL, Erikson H, Ellsinger BM, Mikkelsen KL, Larsson B. Mortality associated with body fat, fat-free mass and body mass index among 60-year-old swedish men-a 22-year follow-up. The study of men born in 1913. Int J Obes Relat Metab Disord. 2000;24: 33–7. 10702748 10.1038/sj.ijo.0801082

[42] Zhu S, Heo M, Plankey M, Faith MS, Allison DB. Associations of body mass index and anthropometric inicators of fat mass and fat free mass with all-cause mortality among women in the first and second National Health and Nutrition Examination Surveys follow-up studies. Annals of Epidemiology. 2003;13:286–93. 12684196 10.1016/s1047-2797(02)00417-9

