# Supplementary material for: Associations between body mass index and mortality or cardiovascular events in a general Korean population
Source: PLoS One. 2017 Sep 15;12(9):e0185024. doi: 10.1371/journal.pone.0185024 (PMC5600387; doi:10.1371/journal.pone.0185024)
Supplement: S1 Table — All HRs were adjusted for age, behavior, income, and family history of cardiovascular disease. Ex-smoker group among women was not presented due to the small number. BMI, body mass index; HTN, hypertension; DM, diabetes mellitus; HR, hazard ratio. (DOCX) [file pone.0185024.s001.docx]

Supplemental Table 1. Multivariate hazard ratios for overall mortality according to body mass index

|  |  | | BMI (kg/m^2^) | | <20 | | 20-22.4 | 22.5-24.9 | | 25-27.4 | | 27.5-29.9 | ≥30 |
| --- | --- | --- | --- | --- | --- | --- | --- | --- | --- | --- | --- | --- | --- |
| **Men** |  | |  | |  | |  |  | |  | |  |  |
| All |  | | N / n | | 17725/1264 | | 49352/2075 | 71313/2086 | | 53063/1271 | | 19371/425 | 7564/136 |
|  |  | | HR | | **1.91** | | **1.40** | **1.10** | | 1 (ref) | | 1.09 | **1.24** |
|  |  | | (95% CI) | | (1.76-2.07) | | (1.31-1.51) | (1.02-1.18) | |  | | (0.98-1.22) | (1.04-1.48) |
| Smoking | Non-smoker | | N / n | | 6115/436 | | 19616/827 | 31139/962 | | 23822/595 | | 8542/202 | 3091/73 |
|  | (never, ex-) | | HR | | **1.97** | | **1.42** | **1.14** | | 1 (ref) | | 1.08 | **1.42** |
|  |  | | (95% CI) | | (1.74-2.23) | | (1.28-1.58) | (1.03-1.26) | |  | | (0.92-1.27) | (1.11-1.81) |
|  | Never smoker | | N / n | | 5017/385 | | 15444/706 | 24020/792 | | 18057/505 | | 6458/168 | 2282/67 |
|  |  | | HR | | **1.86** | | **1.36** | 1.07 | | 1 (ref) | | 1.06 | **1.51** |
|  |  | | (95% CI) | | (1.62-2.13) | | (1.21-1.52) | (0.96-1.2) | |  | | (0.89-1.26) | (1.17-1.94) |
|  | Ex-smoker | | N / n | | 1098/51 | | 4172/121 | 7119/170 | | 5765/90 | | 2084/34 | 809/6 |
|  |  | | HR | | **1.92** | | **1.56** | **1.42** | | 1 (ref) | | 1.22 | 0.85 |
|  |  | | (95% CI) | | (1.35-2.72) | | (1.19-2.06) | (1.1-1.83) | |  | | (0.82-1.81) | (0.37-1.95) |
|  | Current Smoker | | N / n | | 10143/687 | | 25228/1009 | 33116/873 | | 23654/518 | | 8936/170 | 3878/53 |
|  |  | | HR | | **1.80** | | **1.35** | 1.04 | | 1 (ref) | | 1.11 | 1.14 |
|  |  | | (95% CI) | | (1.61-2.02) | | (1.22-1.50) | (0.93-1.16) | |  | | (0.93-1.32) | (0.86-1.52) |
| HTN | No | | N / n | | 14112/659 | | 37663/976 | 49964/903 | | 33413/470 | | 10788/140 | 3637/40 |
|  |  | | HR | | **2.00** | | **1.43** | **1.14** | | 1 (ref) | | 1.16 | 1.29 |
|  |  | | (95% CI) | | (1.77-2.25) | | (1.28-1.60) | (1.02-1.28) | |  | | (0.96-1.40) | (0.93-1.78) |
|  | Yes | | N / n | | 3613/605 | | 11689/1099 | 21349/1183 | | 19650/801 | | 8583/285 | 3927/96 |
|  |  | | HR | | **2.15** | | **1.52** | **1.11** | | 1 (ref) | | 1.01 | 1.14 |
|  |  | | (95% CI) | | (1.93-2.40) | | (1.39-1.67) | (1.02-1.22) | |  | | (0.89-1.16) | (0.92-1.41) |
| DM | No | | N / n | | 15796/938 | | 43901/1530 | 62137/1512 | | 45250/907 | | 16042/288 | 6055/92 |
|  |  | | HR | | **1.85** | | **1.37** | 1.08 | | 1 (ref) | | 1.07 | **1.25** |
|  |  | | (95% CI) | | (1.69-2.03) | | (1.26-1.49) | (0.99-1.17) | |  | | (0.93-1.22) | (1.01-1.55) |
|  | Yes | | N / n | | 1929/326 | | 5451/545 | 9176/574 | | 7813/364 | | 3329/137 | 1509/44 |
|  |  | | HR | | **2.44** | | **1.65** | **1.20** | | 1 (ref) | | 1.11 | 1.12 |
|  |  | | (95% CI) | | (2.1-2.84) | | (1.44-1.88) | (1.06-1.37) | |  | | (0.91-1.35) | (0.82-1.54) |
| **Women** |  |  | |  | |  | | |  | |  |  |  |
| All |  | N / n | | 30184/576 | | 58661/999 | | | 56999/1066 | | 31946/683 | 13210/314 | 6408/163 |
|  |  | HR | | **1.50** | | **1.24** | | | 1.04 | | 1 (ref) | 1.07 | **1.38** |
|  |  | (95% CI) | | (1.34-1.68) | | (1.12-1.36) | | | (0.95-1.15) | |  | (0.93-1.22) | (1.16-1.64) |
| Smoking | Non-smoker | N / n | | 27598/489 | | 54213/875 | | | 52957/956 | | 29676/620 | 12272/285 | 5850/150 |
|  | (never, ex-) | HR | | **1.52** | | **1.22** | | | 1.03 | | 1 (ref) | 1.05 | **1.39** |
|  |  | (95% CI) | | (1.35-1.72) | | (1.1-1.35) | | | (0.93-1.14) | |  | (0.91-1.21) | (1.17-1.67) |
|  | Never smoker | N / n | | 27098/485 | | 53565/867 | | | 52416/948 | | 29400/617 | 12141/284 | 5770/150 |
|  |  | HR | | **1.52** | | **1.21** | | | 1.03 | | 1 (ref) | 1.05 | **1.40** |
|  |  | (95% CI) | | (1.35-1.72) | | (1.09-1.34) | | | (0.93-1.14) | |  | (0.92-1.21) | (1.17-1.67) |
|  | Current smoker | N / n | | 1539/60 | | 2333/80 | | | 1861/58 | | 1066/35 | 458/12 | 318/4 |
|  |  | HR | | 1.25 | | 1.38 | | | 1.18 | | 1 (ref) | 0.98 | 0.72 |
|  |  | (95% CI) | | (0.82-1.90) | | (0.93-2.06) | | | (0.77-1.79) | |  | (0.51-1.88) | (0.26-2.04) |
| HTN | No | N / n | | 26719/264 | | 48499/459 | | | 41262/400 | | 19733/215 | 7025/96 | 2982/31 |
|  |  | HR | | **1.45** | | **1.29** | | | 1.04 | | 1 (ref) | **1.33** | 1.32 |
|  |  | (95% CI) | | (1.21-1.74) | | (1.09-1.51) | | | (0.88-1.22) | |  | (1.04-1.69) | (0.91-1.93) |
|  | Yes | N / n | | 3465/312 | | 10162/540 | | | 15737/666 | | 12213/468 | 6185/218 | 3426/132 |
|  |  | HR | | **1.80** | | **1.31** | | | 1.09 | | 1 (ref) | 0.95 | **1.30** |
|  |  | (95% CI) | | (1.56-2.08) | | (1.16-1.48) | | | (0.97-1.23) | |  | (0.81-1.11) | (1.07-1.58) |
| DM | No | N / n | | 27763/432 | | 52957/760 | | | 48957/705 | | 25970/454 | 10241/222 | 4676/84 |
|  |  | HR | | **1.51** | | **1.27** | | | 0.99 | | 1 (ref) | **1.21** | 1.22 |
|  |  | (95% CI) | | (1.32-1.72) | | (1.13-1.43) | | | (0.88-1.12) | |  | (1.03-1.42) | (0.97-1.54) |
|  | Yes | N / n | | 2421/144 | | 5704/239 | | | 8042/361 | | 5976/229 | 2969/92 | 1732/79 |
|  |  | HR | | **1.72** | | **1.26** | | | **1.20** | | 1 (ref) | 0.80 | **1.48** |
|  |  | (95% CI) | | (1.39-2.11) | | (1.05-1.51) | | | (1.02-1.42) | |  | (0.63-1.02) | (1.14-1.91) |

All HRs were adjusted for age, behavior, income, and family history of cardiovascular disease. Ex-smoker group among women was not presented due to the small number. BMI, body mass index; HTN, hypertension; DM, diabetes mellitus; HR, hazard ra
