# Supplementary material for: Associations between body mass index and mortality or cardiovascular events in a general Korean population
Source: PLoS One. 2017 Sep 15;12(9):e0185024. doi: 10.1371/journal.pone.0185024 (PMC5600387; doi:10.1371/journal.pone.0185024)
Supplement: S2 Table — All HRs were adjusted for age, behavior, income, and family history of cardiovascular disease. Ex-smoker group among women was not presented due to the small number. BMI, body mass index; HTN, hypertension; DM, diabetes mellitus; HR, hazard ratio. (DOCX) [file pone.0185024.s002.docx]

Supplemental Table 2. Multivariate hazard ratios for overall mortality according to body mass index, excluding subjects who died within less than 3 or 5 years after the baseline examination

|  |  |  |  |  | Exclusion of subjects who died within less than 3 years | | | | | |  | Exclusion of subjects who died within less than 5 years | | | | | |
| --- | --- | --- | --- | --- | --- | --- | --- | --- | --- | --- | --- | --- | --- | --- | --- | --- | --- |
|  |  |  | BMI (kg/m^2^) |  | <20 | 20-22.4 | 22.5-24.9 | 25-27.4 | 27.5-29.9 | ≥30 |  | <20 | 20-22.4 | 22.5-24.9 | 25-27.4 | 27.5-29.9 | ≥30 |
|  | **Men** |  |  |  |  |  |  |  |  |  |  |  |  |  |  |  |  |
|  | All |  | N / n |  | 17334/873 | 48795/1518 | 70797/1570 | 52758/966 | 19267/321 | 7526/98 |  | 17021/560 | 48309/1032 | 70309/1082 | 52473/681 | 19164/218 | 7501/73 |
|  |  |  | HR |  | **1.75** | **1.35** | **1.08** | 1 (ref) | 1.10 | **1.23** |  | **1.62** | **1.30** | 1.06 | 1 (ref) | 1.07 | **1.35** |
|  |  |  | (95% CI) |  | (1.59-1.92) | (1.24-1.46) | (1-1.17) |  | (0.97-1.25) | (1-1.51) |  | (1.44-1.81) | (1.18-1.43) | (0.96-1.17) |  | (0.92-1.25) | (1.06-1.71) |
|  | Smoking | Non-smoker | N / n |  | 5974/295 | 19392/603 | 30892/715 | 23681/454 | 8494/154 | 3070/52 |  | 5871/192 | 19187/398 | 30674/497 | 23529/302 | 8439/99 | 3058/40 |
|  |  |  | HR |  | **1.69** | **1.32** | 1.09 | 1 (ref) | 1.10 | **1.38** |  | **1.71** | **1.33** | 1.14 | 1 (ref) | 1.07 | **1.66** |
|  |  |  | (95% CI) |  | (1.46-1.96) | (1.17-1.5) | (0.97-1.22) |  | (0.91-1.32) | (1.03-1.83) |  | (1.42-2.05) | (1.14-1.54) | (0.98-1.31) |  | (0.85-1.34) | (1.19-2.31) |
|  |  | Never smoker | N / n |  | 4896/264 | 15259/521 | 23806/578 | 17946/394 | 6419/129 | 2264/49 |  | 4807/175 | 15082/344 | 23632/404 | 17818/266 | 6371/81 | 2252/37 |
|  |  | (never, ex-) | HR |  | **1.67** | **1.28** | 1.00 | 1 (ref) | 1.06 | **1.46** |  | **1.69** | **1.27** | 1.04 | 1 (ref) | 0.99 | **1.70** |
|  |  |  | (95% CI) |  | (1.42-1.96) | (1.13-1.47) | (0.88-1.14) |  | (0.87-1.29) | (1.09-1.97) |  | (1.39-2.05) | (1.08-1.49) | (0.89-1.21) |  | (0.77-1.27) | (1.21-2.4) |
|  |  | Past smoker | N / n |  | 1078/31 | 4133/82 | 7086/137 | 5735/60 | 2075/25 | 806/3 |  | 1064/17 | 4105/54 | 7042/93 | 5711/36 | 2068/18 | 806/3 |
|  |  |  | HR |  | **1.76** | **1.62** | **1.69** | 1 (ref) | 1.38 | 0.72 |  | 1.68 | **1.83** | **1.92** | 1 (ref) | **1.70** | 1.29 |
|  |  |  | (95% CI) |  | (1.14-2.74) | (1.16-2.26) | (1.24-2.29) |  | (0.86-2.2) | (0.22-2.3) |  | (0.94-3.02) | (1.2-2.8) | (1.3-2.83) |  | (0.96-3) | (0.4-4.22) |
|  |  | Current smoker | N / n |  | 9936/480 | 24958/739 | 32908/665 | 23531/395 | 8890/124 | 3861/36 |  | 9771/315 | 24736/517 | 32696/453 | 23428/292 | 8851/85 | 3851/26 |
|  |  |  | HR |  | **1.74** | **1.33** | 1.05 | 1 (ref) | 1.07 | 1.07 |  | **1.57** | **1.26** | 0.96 | 1 (ref) | 1.00 | 1.09 |
|  |  |  | (95% CI) |  | (1.52-2) | (1.17-1.5) | (0.92-1.18) |  | (0.88-1.31) | (0.76-1.5) |  | (1.33-1.84) | (1.09-1.45) | (0.83-1.11) |  | (0.79-1.28) | (0.73-1.62) |
|  | HTN | No | N / n |  | 13906/453 | 37406/719 | 49742/681 | 33279/336 | 10755/107 | 3624/27 |  | 13750/297 | 37196/509 | 49521/460 | 33190/247 | 10713/65 | 3616/19 |
|  |  |  | HR |  | **1.86** | **1.44** | **1.20** | 1 (ref) | **1.29** | 1.28 |  | **1.69** | **1.40** | 1.11 | 1 (ref) | 1.08 | 1.27 |
|  |  |  | (95% CI) |  | (1.61-2.15) | (1.27-1.64) | (1.05-1.37) |  | (1.03-1.6) | (0.86-1.89) |  | (1.42-2.01) | (1.2-1.63) | (0.95-1.29) |  | (0.82-1.42) | (0.79-2.02) |
|  |  | Yes | N / n |  | 3428/420 | 11389/799 | 21055/889 | 19479/630 | 8512/214 | 3902/71 |  | 3271/263 | 11113/523 | 20788/622 | 19283/434 | 8451/153 | 3885/54 |
|  |  |  | HR |  | **1.96** | **1.42** | 1.06 | 1 (ref) | 0.97 | 1.11 |  | **1.81** | **1.34** | 1.07 | 1 (ref) | 1.02 | 1.27 |
|  |  |  | (95% CI) |  | (1.72-2.23) | (1.27-1.57) | (0.96-1.17) |  | (0.83-1.14) | (0.87-1.42) |  | (1.54-2.12) | (1.18-1.52) | (0.95-1.21) |  | (0.85-1.22) | (0.96-1.69) |
|  | DM | No | N / n |  | 15517/659 | 43495/1124 | 61765/1140 | 45042/699 | 15969/215 | 6025/62 |  | 15285/427 | 43150/779 | 61423/798 | 44844/501 | 15908/154 | 6005/42 |
|  |  |  | HR |  | **1.66** | **1.29** | 1.05 | 1 (ref) | 1.05 | 1.15 |  | **1.52** | **1.24** | 1.02 | 1 (ref) | 1.06 | 1.12 |
|  |  |  | (95% CI) |  | (1.49-1.86) | (1.17-1.42) | (0.95-1.15) |  | (0.9-1.23) | (0.88-1.49) |  | (1.33-1.74) | (1.11-1.39) | (0.92-1.15) |  | (0.89-1.27) | (0.81-1.53) |
|  |  | Yes | N / n |  | 1817/214 | 5300/394 | 9032/430 | 7716/267 | 3298/106 | 1501/36 |  | 1736/133 | 5159/253 | 8886/284 | 7629/180 | 3256/64 | 1496/31 |
|  |  |  | HR |  | **2.35** | **1.67** | **1.24** | 1 (ref) | 1.17 | 1.29 |  | **2.29** | **1.63** | **1.22** | 1 (ref) | 1.05 | **1.71** |
|  |  |  | (95% CI) |  | (1.95-2.82) | (1.43-1.96) | (1.06-1.44) |  | (0.93-1.46) | (0.91-1.82) |  | (1.82-2.88) | (1.34-1.98) | (1.01-1.47) |  | (0.79-1.4) | (1.17-2.51) |
|  | **Women** |  |  |  |  |  |  |  |  |  |  |  |  |  |  |  |  |
|  | All |  | N / n |  | 30018/410 | 58373/711 | 56765/832 | 31770/507 | 13136/240 | 6369/124 |  | 29881/273 | 58162/500 | 56521/588 | 31636/373 | 13069/173 | 6333/88 |
|  |  |  | HR |  | **1.49** | **1.21** | 1.10 | 1 (ref) | 1.10 | **1.45** |  | **1.40** | **1.18** | 1.07 | 1 (ref) | 1.08 | **1.43** |
|  |  |  | (95% CI) |  | (1.31-1.7) | (1.08-1.36) | (0.99-1.23) |  | (0.94-1.28) | (1.19-1.76) |  | (1.2-1.65) | (1.03-1.35) | (0.94-1.21) |  | (0.9-1.29) | (1.13-1.8) |
|  | Smoking | Non-smoker | N / n |  | 27460/351 | 53960/622 | 52746/745 | 29516/460 | 12204/217 | 5815/115 |  | 27349/240 | 53780/442 | 52525/524 | 29394/338 | 12144/157 | 5781/81 |
|  |  | (never, ex-) | HR |  | **1.53** | **1.19** | 1.09 | 1 (ref) | 1.08 | **1.47** |  | **1.47** | **1.17** | 1.04 | 1 (ref) | 1.06 | **1.43** |
|  |  |  | (95% CI) |  | (1.33-1.76) | (1.05-1.34) | (0.97-1.22) |  | (0.92-1.27) | (1.2-1.8) |  | (1.24-1.73) | (1.01-1.35) | (0.91-1.2) |  | (0.88-1.28) | (1.12-1.82) |
|  |  | Never smoker | N / n |  | 26962/349 | 53316/618 | 52207/739 | 29243/460 | 12073/216 | 5735/115 |  | 26851/238 | 53136/438 | 51989/521 | 29121/338 | 12013/156 | 5701/81 |
|  |  |  | HR |  | **1.52** | **1.18** | 1.08 | 1 (ref) | 1.07 | **1.46** |  | **1.45** | **1.16** | 1.04 | 1 (ref) | 1.05 | **1.43** |
|  |  |  | (95% CI) |  | (1.32-1.75) | (1.04-1.33) | (0.96-1.21) |  | (0.91-1.26) | (1.19-1.8) |  | (1.23-1.72) | (1-1.33) | (0.91-1.19) |  | (0.87-1.27) | (1.12-1.82) |
|  |  | Current smoker | N / n |  | 1521/42 | 2305/52 | 1844/41 | 1057/26 | 454/8 | 316/2 |  | 1501/22 | 2289/36 | 1831/28 | 1050/19 | 453/7 | 316/2 |
|  |  |  | HR |  | 1.40 | **1.30** | 1.18 | 1 (ref) | 0.85 | 0.49 |  | 1.12 | 1.27 | 1.14 | 1 (ref) | 1.01 | 0.78 |
|  |  |  | (95% CI) |  | (0.85-2.32) | (0.81-2.1) | (0.72-1.93) |  | (0.38-1.89) | (0.12-2.07) |  | (0.6-2.1) | (0.73-2.23) | (0.63-2.05) |  | (0.42-2.41) | (0.18-3.35) |
|  | HTN | No | N / n |  | 26647/192 | 48371/331 | 41173/311 | 19671/153 | 6999/70 | 2976/25 |  | 26584/129 | 48264/224 | 41094/232 | 19636/118 | 6977/48 | 2967/16 |
|  |  |  | HR |  | **1.51** | **1.33** | 1.14 | 1 (ref) | **1.37** | **1.55** |  | **1.36** | 1.21 | 1.11 | 1 (ref) | 1.22 | 1.32 |
|  |  |  | (95% CI) |  | (1.22-1.87) | (1.1-1.62) | (0.94-1.38) |  | (1.03-1.82) | (1.01-2.36) |  | (1.05-1.75) | (0.97-1.52) | (0.89-1.39) |  | (0.87-1.71) | (0.78-2.23) |
|  |  | Yes | N / n |  | 3371/218 | 10002/380 | 15592/521 | 12099/354 | 6137/170 | 3393/99 |  | 3297/144 | 9898/276 | 15427/356 | 12000/255 | 6092/125 | 3366/72 |
|  |  |  | HR |  | **1.74** | **1.23** | 1.13 | 1 (ref) | 0.97 | **1.31** |  | **1.66** | **1.25** | 1.07 | 1 (ref) | 1.00 | **1.36** |
|  |  |  | (95% CI) |  | (1.47-2.07) | (1.07-1.43) | (0.99-1.29) |  | (0.81-1.17) | (1.05-1.64) |  | (1.35-2.05) | (1.06-1.49) | (0.91-1.26) |  | (0.8-1.23) | (1.04-1.76) |
|  | DM | No | N / n |  | 27640/309 | 52745/548 | 48810/558 | 25851/335 | 10190/171 | 4653/61 |  | 27532/201 | 52591/394 | 48650/398 | 25768/252 | 10146/127 | 4634/42 |
|  |  |  | HR |  | **1.49** | **1.26** | 1.07 | 1 (ref) | **1.27** | 1.22 |  | **1.34** | **1.24** | 1.02 | 1 (ref) | **1.25** | 1.14 |
|  |  |  | (95% CI) |  | (1.27-1.74) | (1.1-1.45) | (0.93-1.22) |  | (1.05-1.52) | (0.93-1.6) |  | (1.11-1.62) | (1.06-1.46) | (0.87-1.2) |  | (1.01-1.55) | (0.82-1.58) |
|  |  | Yes | N / n |  | 2378/101 | 5628/163 | 7955/274 | 5919/172 | 2946/69 | 1716/63 |  | 2349/72 | 5571/106 | 7871/190 | 5868/121 | 2923/46 | 1699/46 |
|  |  |  | HR |  | **1.80** | 1.19 | **1.23** | 1 (ref) | 0.79 | **1.59** |  | **1.91** | 1.10 | 1.21 | 1 (ref) | 0.74 | **1.69** |
|  |  |  | (95% CI) |  | (1.4-2.31) | (0.96-1.47) | (1.02-1.49) |  | (0.6-1.05) | (1.19-2.12) |  | (1.42-2.57) | (0.85-1.43) | (0.97-1.53) |  | (0.53-1.04) | (1.21-2.38) |

All HRs were adjusted for age, behavior, income, and family history of cardiovascular disease. Ex-smoker group among women was not presented due to the small number. BMI, body mass index; HTN, hypertension; DM, diabetes mellitus; HR, hazard ratio.
