# Supplementary material for: Associations between body mass index and mortality or cardiovascular events in a general Korean population
Source: PLoS One. 2017 Sep 15;12(9):e0185024. doi: 10.1371/journal.pone.0185024 (PMC5600387; doi:10.1371/journal.pone.0185024)
Supplement: S3 Table — All HRs were adjusted for age, behavior, income, and family history of cardiovascular disease. BMI, body mass index; CVD, cardiovascular disease; HR, hazard rati. (DOCX) [file pone.0185024.s003.docx]

Supplemental Table 3. Multivariate hazard ratios for cardiovascular disease mortality and a cardiovascular disease event according to body mass index

|  | BMI (kg/m^2^) | <20 | 20-22.4 | 22.5-24.9 | 25-27.4 | 27.5-29.9 | ≥30 |
| --- | --- | --- | --- | --- | --- | --- | --- |
| **Mortality** |  |  |  |  |  |  |  |
| **Men** | N | 17725 | 49352 | 71313 | 53063 | 19371 | 7564 |
| CVD | n | 129 | 254 | 262 | 164 | 59 | 14 |
|  | HR | **1.35** | **1.25** | 1.04 | 1 (ref) | 1.20 | 1.01 |
|  | (95% CI) | (1.07-1.71) | (1.03-1.53) | (0.86-1.27) |  | (0.89-1.62) | (0.59-1.74) |
| Ischemic heart disease | n | 46 | 107 | 103 | 64 | 27 | 7 |
|  | HR | 1.31 | 1.40 | 1.06 | 1 (ref) | 1.38 | 1.25 |
|  | (95% CI) | (0.89-1.94) | (1.02-1.91) | (0.78-1.45) |  | (0.88-2.17) | (0.57-2.73) |
| Ischemic stroke | n | 23 | 42 | 47 | 20 | 9 | 3 |
|  | HR | 1.61 | 1.51 | 1.44 | 1 (ref) | 1.64 | 2.07 |
|  | (95% CI) | (0.87-2.98) | (0.88-2.59) | (0.85-2.44) |  | (0.74-3.60) | (0.62-6.98) |
| Hemorrhagic stroke | n | 33 | 57 | 52 | 50 | 15 | 3 |
|  | HR | 1.42 | 1.04 | 0.72 | 1 (ref) | 0.94 | 0.64 |
|  | (95% CI) | (0.9-2.23) | (0.71-1.53) | (0.49-1.06) |  | (0.53-1.68) | (0.20-2.04) |
| **Women** | N | 30184 | 58661 | 56999 | 31946 | 13210 | 6408 |
| CVD | n | 97 | 155 | 165 | 112 | 42 | 29 |
|  | HR | **1.51** | 1.19 | 1.00 | 1 (ref) | 0.86 | **1.50** |
|  | (95% CI) | (1.15-1.99) | (0.93-1.52) | (0.79-1.27) |  | (0.60-1.23) | (1.00-2.26) |
| Ischemic heart disease | n | 21 | 40 | 40 | 36 | 8 | 11 |
|  | HR | 0.91 | 0.94 | 0.76 | 1 (ref) | 0.52 | 1.82 |
|  | (95% CI) | (0.52-1.57) | (0.6-1.47) | (0.48-1.19) |  | (0.24-1.11) | (0.93-3.58) |
| Ischemic stroke | n | 27 | 25 | 34 | 27 | 7 | 11 |
|  | HR | 1.68 | 0.81 | 0.87 | 1 (ref) | 0.60 | **2.36** |
|  | (95% CI) | (0.98-2.88) | (0.47-1.39) | (0.52-1.44) |  | (0.26-1.37) | (1.17-4.76) |
| Hemorrhagic stroke | n | 25 | 54 | 54 | 23 | 17 | 5 |
|  | HR | **2.04** | **1.98** | 1.56 | 1 (ref) | 1.70 | 1.22 |
|  | (95% CI) | (1.15-3.63) | (1.21-3.23) | (0.95-2.54) |  | (0.91-3.18) | (0.46-3.21) |
| **Event** |  |  |  |  |  |  |  |
| **Men** | N | 17725 | 49352 | 71313 | 53063 | 19371 | 7564 |
| CVD | n | 1244 | 2873 | 4252 | 3416 | 1185 | 423 |
|  | HR | **1.08** | 1 (ref) | **1.07** | **1.23** | **1.34** | **1.67** |
|  | (95% CI) | (1.01-1.16) |  | (1.02-1.12) | (1.17-1.29) | (1.25-1.43) | (1.51-1.85) |
| Ischemic heart disease | n | 784 | 1832 | 2817 | 2289 | 821 | 297 |
|  | HR | **1.10** | 1 (ref) | **1.10** | **1.26** | **1.41** | **1.77** |
|  | (95% CI) | (1.01-1.2) |  | (1.03-1.16) | (1.18-1.34) | (1.3-1.53) | (1.57-2) |
| Ischemic stroke | n | 358 | 854 | 1150 | 898 | 297 | 103 |
|  | HR | 0.97 | 1 (ref) | 1.02 | **1.18** | **1.26** | **1.60** |
|  | (95% CI) | (0.85-1.09) |  | (0.93-1.11) | (1.07-1.29) | (1.1-1.44) | (1.3-1.97) |
| Hemorrhagic stroke | n | 143 | 284 | 375 | 324 | 94 | 38 |
|  | HR | **1.28** | 1 (ref) | 0.95 | 1.16 | 1.03 | 1.38 |
|  | (95% CI) | (1.04-1.56) |  | (0.81-1.11) | (0.98-1.36) | (0.81-1.30) | (0.98-1.94) |
| **Women** | N | 30184 | 58661 | 56999 | 31946 | 13210 | 6408 |
| CVD | n | 905 | 2337 | 3415 | 2483 | 1201 | 583 |
|  | HR | **0.93** | 1 (ref) | **1.15** | **1.28** | **1.49** | **1.76** |
|  | (95% CI) | (0.86-1.00) |  | (1.09-1.21) | (1.21-1.35) | (1.39-1.60) | (1.61-1.93) |
| Ischemic heart disease | n | 491 | 1295 | 2013 | 1613 | 729 | 382 |
|  | HR | 0.93 | 1 (ref) | **1.22** | **1.49** | **1.61** | **2.06** |
|  | (95% CI) | (0.84-1.03) |  | (1.14-1.31) | (1.39-1.61) | (1.47-1.76) | (1.84-2.31) |
| Ischemic stroke | n | 255 | 650 | 901 | 643 | 349 | 174 |
|  | HR | 0.88 | 1 (ref) | 1.05 | 1.10 | **1.40** | **1.72** |
|  | (95% CI) | (0.76-1.02) |  | (0.95-1.16) | (0.98-1.22) | (1.23-1.6) | (1.45-2.03) |
| Hemorrhagic stroke | n | 112 | 252 | 339 | 186 | 103 | 53 |
|  | HR | 1.07 | 1 (ref) | 1.07 | 0.90 | 1.18 | **1.45** |
|  | (95% CI) | (0.85-1.34) |  | (0.91-1.26) | (0.74-1.09) | (0.94-1.48) | (1.08-1.96) |

All HRs were adjusted for age, behavior, income, and family history of cardiovascular disease. BMI, body mass index; CVD, cardiovascular disease; HR, hazard ratio.
