# Supplementary material for: Associations between body mass index and mortality or cardiovascular events in a general Korean population
Source: PLoS One. 2017 Sep 15;12(9):e0185024. doi: 10.1371/journal.pone.0185024 (PMC5600387; doi:10.1371/journal.pone.0185024)
Supplement: S4 Table — All HRs were adjusted for age, behavior, income, and family history of cardiovascular disease. BMI, body mass index; CVD, cardiovascular disease; HR, hazard ratio. (DOCX) [file pone.0185024.s004.docx]

Supplemental Table 4. Multivariate hazard ratios for cardiovascular disease mortality and a cardiovascular disease event according to body mass index, excluding subjects who died within less than 3 years after baseline examination

|  | BMI (kg/m^2^) | <20 | 20-22.4 | 22.5-24.9 | 25-27.4 | 27.5-29.9 | ≥30 |
| --- | --- | --- | --- | --- | --- | --- | --- |
| **Mortality** |  |  |  |  |  |  |  |
| **Men** |  |  |  |  |  |  |  |
| CVD | N / n | 17334/94 | 48795/192 | 70797/199 | 52758/131 | 19267/41 | 7526/11 |
|  | HR | 1.25 | 1.18 | 0.99 | 1 (ref) | 1.06 | 1.04 |
|  | (95% CI) | (0.95 -1.64 ) | (0.95 -1.48 ) | (0.79 -1.23 ) |  | (0.75 -1.51 ) | (0.56 -1.92 ) |
| Ischemic heart disease | N / n | 17334/35 | 48795/79 | 70797/79 | 52758/51 | 19267/19 | 7526/6 |
|  | HR | 1.26 | 1.29 | 1.02 | 1 (ref) | 1.24 | 1.4 |
|  | (95% CI) | (0.81 -1.96 ) | (0.9 -1.84 ) | (0.72 -1.45 ) |  | (0.73 -2.11 ) | (0.6 -3.27 ) |
| Ischemic stroke | N / n | 17334/18 | 48795/33 | 70797/33 | 52758/18 | 19267/7 | 7526/2 |
|  | HR | 1.43 | 1.33 | 1.12 | 1 (ref) | 1.42 | 1.59 |
|  | (95% CI) | (0.73 -2.81 ) | (0.74 -2.37 ) | (0.63 -2 ) |  | (0.59 -3.4 ) | (0.37 -6.86 ) |
| Hemorrhagic stroke | N / n | 17334/20 | 48795/42 | 70797/34 | 52758/38 | 19267/8 | 7526/2 |
|  | HR | 1.19 | 1.03 | **0.62** | 1 (ref) | 0.67 | 0.58 |
|  | (95% CI) | (0.68 -2.08 ) | (0.66 -1.6 ) | (0.39 -0.99 ) |  | (0.31 -1.43 ) | (0.14 -2.4 ) |
| **Women** |  |  |  |  |  |  |  |
| CVD | N / n | 30018/76 | 58373/108 | 56765/126 | 31770/85 | 13136/27 | 6369/23 |
|  | HR | **1.63** | 1.12 | 1.01 | 1 (ref) | 0.73 | **1.6** |
|  | (95% CI) | (1.19 -2.23 ) | (0.84 -1.49 ) | (0.77 -1.33 ) |  | (0.47 -1.12 ) | (1.01 -2.53 ) |
| Ischemic heart disease | N / n | 30018/18 | 58373/29 | 56765/28 | 31770/31 | 13136/7 | 6369/6 |
|  | HR | 0.95 | 0.8 | 0.62 | 1 (ref) | 0.53 | 1.18 |
|  | (95% CI) | (0.52 -1.71 ) | (0.48 -1.33 ) | (0.37 -1.04 ) |  | (0.23 -1.2 ) | (0.49 -2.83 ) |
| Ischemic stroke | N / n | 30018/20 | 58373/17 | 56765/30 | 31770/20 | 13136/3 | 6369/11 |
|  | HR | **1.85** | 0.77 | 1.04 | 1 (ref) | 0.34 | **3.22** |
|  | (95% CI) | (0.99 -3.47 ) | (0.4 -1.47 ) | (0.59 -1.83 ) |  | (0.1 -1.16 ) | (1.54 -6.73 ) |
| Hemorrhagic stroke | N / n | 30018/21 | 58373/35 | 56765/37 | 31770/12 | 13136/10 | 6369/4 |
|  | HR | **3.37** | **2.5** | **2.05** | 1 (ref) | 1.91 | 1.90 |
|  | (95% CI) | (1.64 -6.91 ) | (1.3 -4.84 ) | (1.07 -3.94 ) |  | (0.83 -4.42 ) | (0.61 -5.88 ) |
| **Event** |  |  |  |  |  |  |  |
| **Men** |  |  |  |  |  |  |  |
| CVD | N / n | 16991/804 | 48006/1952 | 69598/2917 | 51830/2410 | 18910/799 | 7392/280 |
|  | HR | 1.06 | 1 (ref) | **1.07** | **1.26** | **1.33** | **1.69** |
|  | (95% CI) | (0.97 -1.15 ) |  | (1.01 -1.13 ) | (1.19 -1.34 ) | (1.23 -1.45 ) | (1.49 -1.91 ) |
| Ischemic heart disease | N / n | 17127/519 | 48302/1252 | 70025/1965 | 52141/1624 | 19017/554 | 7438/203 |
|  | HR | 1.09 | 1 (ref) | **1.11** | **1.30** | **1.40** | **1.83** |
|  | (95% CI) | (0.98 -1.21 ) |  | (1.04 -1.19 ) | (1.21 -1.4 ) | (1.27 -1.55 ) | (1.58 -2.13 ) |
| Ischemic stroke | N / n | 17236/232 | 48574/600 | 70486/802 | 52504/624 | 19179/205 | 7491/66 |
|  | HR | 0.92 | 1 (ref) | 1 | **1.15** | **1.22** | **1.49** |
|  | (95% CI) | (0.79 -1.07 ) |  | (0.9 -1.11 ) | (1.03 -1.29 ) | (1.04 -1.44 ) | (1.16 -1.93 ) |
| Hemorrhagic stroke | N / n | 17306/93 | 48722/184 | 70702/249 | 52681/228 | 19250/69 | 7512/23 |
|  | HR | **1.32** | 1 (ref) | 0.96 | **1.24** | 1.17 | 1.33 |
|  | (95% CI) | (1.03 -1.69 ) |  | (0.8 -1.17 ) | (1.02 -1.51 ) | (0.88 -1.54 ) | (0.86 -2.05 ) |
| **Women** |  |  |  |  |  |  |  |
| CVD | N / n | 29737/581 | 57672/1548 | 55748/2328 | 31026/1685 | 12746/782 | 6177/374 |
|  | HR | 0.92 | 1 (ref) | **1.17** | **1.29** | **1.46** | **1.75** |
|  | (95% CI) | (0.84 -1.01 ) |  | (1.1 -1.25 ) | (1.2 -1.38 ) | (1.34 -1.6 ) | (1.56 -1.96 ) |
| Ischemic heart disease | N / n | 29856/311 | 57978/858 | 56180/1393 | 31294/1110 | 12912/492 | 6246/248 |
|  | HR | 0.91 | 1 (ref) | **1.26** | **1.53** | **1.62** | **2.05** |
|  | (95% CI) | (0.8 -1.04 ) |  | (1.15 -1.37 ) | (1.4 -1.67 ) | (1.45 -1.82 ) | (1.78 -2.36 ) |
| Ischemic stroke | N / n | 29951/173 | 58184/430 | 56492/607 | 31589/444 | 13022/226 | 6324/125 |
|  | HR | 0.92 | 1 (ref) | 1.06 | 1.13 | **1.36** | **1.89** |
|  | (95% CI) | (0.77 -1.1 ) |  | (0.93 -1.2 ) | (0.99 -1.29 ) | (1.16 -1.6 ) | (1.55 -2.31 ) |
| Hemorrhagic stroke | N / n | 29981/64 | 58305/159 | 56664/215 | 31717/119 | 13110/69 | 6358/39 |
|  | HR | 0.98 | 1 (ref) | 1.07 | 0.9 | 1.23 | **1.71** |
|  | (95% CI) | (0.73 -1.31 ) |  | (0.87 -1.31 ) | (0.71 -1.14 ) | (0.93 -1.64 ) | (1.2 -2.43 ) |

All HRs were adjusted for age, behavior, income, and family history of cardiovascular disease. BMI, body mass index; CVD, cardiovascular disease; HR, hazard ratio.
