# Supplementary material for: Associations between body mass index and mortality or cardiovascular events in a general Korean population
Source: PLoS One. 2017 Sep 15;12(9):e0185024. doi: 10.1371/journal.pone.0185024 (PMC5600387; doi:10.1371/journal.pone.0185024)
Supplement: S5 Table — All HRs were adjusted for age, behavior, income, and family history of cardiovascular disease. Ex-smoker group among women was not presented due to the small number. BMI, body mass index; HTN, hypertension; DM, diabetes mellitus; HR, hazard ratio. (DOCX) [file pone.0185024.s005.docx]

Supplemental Table 5. Multivariate hazard ratios for cardiovascular disease mortality according to body mass index

|  |  | BMI (kg/m^2^) | <20 | 20-22.4 | 22.5-24.9 | 25-27.4 | 27.5-29.9 | ≥30 |
| --- | --- | --- | --- | --- | --- | --- | --- | --- |
| **Men** |  |  |  |  |  |  |  |  |
| All |  | N / n | 17725/129 | 49352/254 | 71313/262 | 53063/164 | 19371/59 | 7564/14 |
|  |  | HR | **1.35** | **1.25** | 1.04 | 1 (ref) | 1.20 | 1.01 |
|  |  | 95% CI | (1.07-1.71) | (1.03-1.53) | (0.86-1.27) |  | (0.89-1.62) | (0.59-1.74) |
| Smoking | Non-smoker | N / n | 6115/35 | 19616/97 | 31139/123 | 23822/84 | 8542/23 | 3091/9 |
|  | (never, ex-) | HR | 1.02 | 1.12 | 1.01 | 1 (ref) | 0.89 | 1.27 |
|  |  | 95% CI | (0.68-1.51) | (0.83-1.5) | (0.76-1.33) |  | (0.56-1.41) | (0.64-2.53) |
|  | Never smoker | N / n | 5017/30 | 15444/83 | 24020/104 | 18057/69 | 6458/21 | 2282/8 |
|  |  | HR | 0.97 | 1.10 | 1.01 | 1 (ref) | 0.98 | 1.36 |
|  |  | 95% CI | (0.63-1.49) | (0.8-1.52) | (0.74-1.36) |  | (0.60-1.61) | (0.65-2.83) |
|  | Ex-smoker | N / n | 1098/5 | 4172/14 | 7119/19 | 5765/15 | 2084/2 | 809/1 |
|  |  | HR | 0.96 | 0.98 | 0.93 | 1 (ref) | 0.43 | 0.82 |
|  |  | 95% CI | (0.34-2.69) | (0.47-2.05) | (0.47-1.84) |  | (0.10-1.90) | (0.11-6.24) |
|  | Current smoker | N / n | 10143/75 | 25228/126 | 33116/117 | 23654/63 | 8936/30 | 3878/3 |
|  |  | HR | **1.46** | 1.32 | 1.12 | 1 (ref) | 1.67 | 0.55 |
|  |  | 95% CI | (1.04-2.05) | (0.97-1.79) | (0.82-1.52) |  | (1.08-2.58) | (0.17-1.74) |
| HTN | No | N / n | 14112/55 | 37663/85 | 49964/67 | 33413/38 | 10788/15 | 3637/3 |
|  |  | HR | **1.92** | **1.49** | 1.03 | 1 (ref) | 1.55 | 1.17 |
|  |  | 95% CI | (1.26-2.91) | (1.01-2.18) | (0.7-1.54) |  | (0.85-2.82) | (0.36-3.80) |
|  | Yes | N / n | 3613/74 | 11689/169 | 21349/195 | 19650/126 | 8583/44 | 3927/11 |
|  |  | HR | **1.53** | **1.41** | 1.14 | 1 (ref) | 1.01 | 0.84 |
|  |  | 95% CI | (1.14-2.05) | (1.11-1.78) | (0.91-1.42) |  | (0.72-1.43) | (0.45-1.55) |
| DM | No | N / n | 15796/96 | 43901/188 | 62137/190 | 45250/112 | 16042/35 | 6055/10 |
|  |  | HR | **1.36** | **1.28** | 1.07 | 1 (ref) | 1.07 | 1.12 |
|  |  | 95% CI | (1.03-1.80) | (1.01-1.62) | (0.84-1.35) |  | (0.73-1.57) | (0.59-2.15) |
|  | Yes | N / n | 1929/33 | 5451/66 | 9176/72 | 7813/52 | 3329/24 | 1509/4 |
|  |  | HR | **1.57** | 1.32 | 1.04 | 1 (ref) | 1.42 | 0.74 |
|  |  | 95% CI | (1.01-2.43) | (0.92-1.90) | (0.73-1.48) |  | (0.87-2.30) | (0.27-2.05) |
| **Women** |  |  |  |  |  |  |  |  |
| All |  | N / n | 30184/97 | 58661/155 | 56999/165 | 31946/112 | 13210/42 | 6408/29 |
|  |  | HR | **1.51** | 1.19 | 1.00 | 1 (ref) | 0.86 | **1.50** |
|  |  | 95% CI | (1.15-1.99) | (0.93-1.52) | (0.79-1.27) |  | (0.60-1.23) | (1.00-2.26) |
| Smoking | Non-smoker | N / n | 27598/83 | 54213/137 | 52957/149 | 29676/105 | 12272/38 | 5850/27 |
|  | (never, ex-) | HR | **1.51** | 1.15 | 0.96 | 1 (ref) | 0.82 | 1.47 |
|  |  | 95% CI | (1.13-2.02) | (0.89-1.49) | (0.75-1.24) |  | (0.56-1.19) | (0.97-2.25) |
|  | Never smoker | N / n | 27098/83 | 53565/135 | 52416/146 | 29400/103 | 12141/38 | 5770/27 |
|  |  | HR | **1.55** | 1.16 | 0.96 | 1 (ref) | 0.83 | 1.49 |
|  |  | 95% CI | (1.16-2.08) | (0.89-1.50) | (0.75-1.24) |  | (0.58-1.21) | (0.98-2.28) |
|  | Current smoker | N / n | 1539/14 | 2333/11 | 1861/10 | 1066/5 | 458/1 | 318/1 |
|  |  | HR | 1.96 | 1.34 | 1.48 | 1 (ref) | 0.60 | 1.39 |
|  |  | 95% CI | (0.70-5.44) | (0.46-3.85) | (0.51-4.34) |  | (0.07-5.12) | (0.16-11.94) |
| HTN | No | N / n | 26719/27 | 48499/47 | 41262/45 | 19733/23 | 7025/10 | 2982/2 |
|  |  | HR | 1.37 | 1.27 | 1.11 | 1 (ref) | 1.29 | 0.82 |
|  |  | 95% CI | (0.78-2.40) | (0.77-2.10) | (0.67-1.84) |  | (0.61-2.71) | (0.19-3.47) |
|  | Yes | N / n | 3465/70 | 10162/108 | 15737/120 | 12213/89 | 6185/32 | 3426/27 |
|  |  | HR | **2.01** | **1.34** | 1.03 | 1 (ref) | 0.73 | 1.42 |
|  |  | 95% CI | (1.46-2.76) | (1.01-1.77) | (0.78-1.36) |  | (0.49-1.1) | (0.92-2.19) |
| DM | No | N / n | 27763/75 | 52957/128 | 48957/109 | 25970/74 | 10241/28 | 4676/11 |
|  |  | HR | **1.58** | **1.35** | 0.96 | 1 (ref) | 0.93 | 0.98 |
|  |  | 95% CI | (1.14-2.18) | (1.01-1.79) | (0.72-1.29) |  | (0.60-1.43) | (0.52-1.85) |
|  | Yes | N / n | 2421/22 | 5704/27 | 8042/56 | 5976/38 | 2969/14 | 1732/18 |
|  |  | HR | 1.49 | 0.85 | 1.12 | 1 (ref) | 0.73 | **2.08** |
|  |  | 95% CI | (0.88-2.53) | (0.52-1.40) | (0.75-1.70) |  | (0.40-1.35) | (1.18-3.64) |

All HRs were adjusted for age, behavior, income, and family history of cardiovascular disease. Ex-smoker group among women was not presented due to the small number. BMI, body mass index; HTN, hypertension; DM, diabetes mellitus; HR, hazard ratio.
