# Supplementary material for: Associations between body mass index and mortality or cardiovascular events in a general Korean population
Source: PLoS One. 2017 Sep 15;12(9):e0185024. doi: 10.1371/journal.pone.0185024 (PMC5600387; doi:10.1371/journal.pone.0185024)
Supplement: S6 Table — All HRs were adjusted for age, behavior, income, and family history of cardiovascular disease. Ex-smoker group among women was not presented due to the small number. BMI, body mass index; HTN, hypertension; DM, diabetes mellitus; HR, hazard ratio. (DOCX) [file pone.0185024.s006.docx]

Supplemental Table 6. Multivariate hazard ratios for cardiovascular disease mortality according to body mass index, excluding subjects who died within less than 3 years after baseline examination

|  |  | BMI (kg/m^2^) | <20 | 20-22.4 | 22.5-24.9 | 25-27.4 | 27.5-29.9 | ≥30 |
| --- | --- | --- | --- | --- | --- | --- | --- | --- |
| **Men** |  |  |  |  |  |  |  |  |
| All |  | N / n | 17334/94 | 48795/192 | 70797/199 | 52758/131 | 19267/41 | 7526/11 |
|  |  | HR | 1.25 | 1.18 | 0.99 | 1 (ref) | 1.06 | 1.04 |
|  |  | 95% CI | (0.95-1.64) | (0.95-1.48) | (0.79-1.23) |  | (0.75-1.51) | (0.56-1.92) |
| Smoking | Non-smoker | N / n | 5974/23 | 19392/68 | 30892/89 | 23681/69 | 8494/16 | 3070/6 |
|  | (never, ex-) | HR | 0.80 | **0.93** | 0.87 | 1 (ref) | 0.76 | 1.07 |
|  |  | 95% CI | (0.50-1.29) | (0.66-1.3) | (0.64-1.19) |  | (0.44-1.31) | (0.47-2.47) |
|  | Never smoker | N / n | 4896/20 | 15259/59 | 23806/73 | 17946/58 | 6419/14 | 2264/5 |
|  |  | HR | 0.80 | 0.94 | 0.84 | 1 (ref) | 0.79 | 1.04 |
|  |  | 95% CI | (0.48-1.34) | (0.65-1.36) | (0.60-1.19) |  | (0.44-1.41) | (0.42-2.59) |
|  | Ex-smoker | N / n | 1078/3 | 4133/9 | 7086/16 | 5735/11 | 2075/2 | 806/1 |
|  |  | HR | 0.76 | 0.84 | 1.02 | 1 (ref) | 0.61 | 1.41 |
|  |  | 95% CI | (0.21-2.78) | (0.34-2.05) | (0.47-2.20) |  | (0.13-2.77) | (0.18-11.06) |
|  | Current smoker | N / n | 9936/57 | 24958/99 | 32908/91 | 23531/50 | 8890/21 | 3861/3 |
|  |  | HR | **1.54** | **1.37** | 1.11 | 1 (ref) | 1.47 | 0.71 |
|  |  | 95% CI | (1.04-2.28) | (0.97-1.93) | (0.79-1.57) |  | (0.89-2.46) | (0.22-2.28) |
| HTN | No | N / n | 13906/40 | 37406/67 | 49742/52 | 33279/30 | 10755/10 | 3624/3 |
|  |  | HR | **1.81** | **1.50** | 1.02 | 1 (ref) | 1.35 | 1.55 |
|  |  | 95% CI | (1.11-2.95) | (0.97-2.31) | (0.65-1.61) |  | (0.66-2.77) | (0.47-5.08) |
|  | Yes | N / n | 3428/54 | 11389/125 | 21055/147 | 19479/101 | 8512/31 | 3902/8 |
|  |  | HR | **1.42** | **1.30** | 1.06 | 1 (ref) | 0.89 | 0.77 |
|  |  | 95% CI | (1.01-2) | (0.99-1.69) | (0.82-1.37) |  | (0.60-1.34) | (0.37-1.58) |
| DM | No | N / n | 15517/71 | 43495/141 | 61765/147 | 45042/91 | 15969/23 | 6025/8 |
|  |  | HR | 1.23 | 1.17 | 1.01 | 1 (ref) | 0.88 | 1.15 |
|  |  | 95% CI | (0.89-1.69) | (0.89-1.52) | (0.78-1.31) |  | (0.56-1.39) | (0.56-2.38) |
|  | Yes | N / n | 1817/23 | 5300/51 | 9032/52 | 7716/40 | 3298/18 | 1501/3 |
|  |  | HR | 1.53 | 1.38 | 0.97 | 1 (ref) | 1.37 | 0.73 |
|  |  | 95% CI | (0.9-2.57) | (0.91-2.10) | (0.64-1.47) |  | (0.79-2.40) | (0.23-2.38) |
| **Women** |  |  |  |  |  |  |  |  |
| All |  | N / n | 30018/76 | 58373/108 | 56765/126 | 31770/85 | 13136/27 | 6369/23 |
|  |  | HR | **1.63** | 1.12 | 1.01 | 1 (ref) | 0.73 | **1.60** |
|  |  | 95% CI | (1.19-2.23) | (0.84-1.49) | (0.77-1.33) |  | (0.47-1.12) | (1.01-2.53) |
| Smoking | Non-smoker | N / n | 27460/64 | 53960/96 | 52746/112 | 29516/81 | 12204/24 | 5815/23 |
|  | (never, ex-) | HR | **1.57** | **1.06** | 0.94 | 1 (ref) | 0.67 | **1.63** |
|  |  | 95% CI | (1.12-2.18) | (0.79-1.42) | (0.71-1.25) |  | (0.42-1.05) | (1.03-2.60) |
|  | Never smoker | N / n | 26962/64 | 53316/95 | 52207/110 | 29243/81 | 12073/24 | 5735/23 |
|  |  | HR | **1.57** | 1.05 | 0.92 | 1 (ref) | 0.67 | **1.63** |
|  |  | 95% CI | (1.13-2.19) | (0.78-1.41) | (0.69-1.23) |  | (0.42-1.05) | (1.03-2.59) |
|  | Current smoker | N / n | 1521/12 | 2305/7 | 1844/8 | 1057/2 | 454/1 | 316/0 |
|  |  | HR | **5.31** | **2.42** | 3.18 | 1 (ref) | 1.31 | - |
|  |  | 95% CI | (1.15-24.46) | (0.5-11.79) | (0.66-15.26) |  | (0.12-14.69) | - |
| HTN | No | N / n | 26647/24 | 48371/34 | 41173/37 | 19671/17 | 6999/7 | 2976/2 |
|  |  | HR | 1.61 | 1.29 | 1.25 | 1 (ref) | 1.21 | 1.11 |
|  |  | 95% CI | (0.86-3.03) | (0.72-2.32) | (0.71-2.23) |  | (0.50-2.91) | (0.26-4.79) |
|  | Yes | N / n | 3371/52 | 10002/74 | 15592/89 | 12099/68 | 6137/20 | 3393/21 |
|  |  | HR | **2.12** | 1.23 | 1.01 | 1 (ref) | 0.59 | 1.46 |
|  |  | 95% CI | (1.46-3.06) | (0.88-1.71) | (0.73-1.38) |  | (0.36-0.98) | (0.89-2.38) |
| DM | No | N / n | 27640/56 | 52745/88 | 48810/82 | 25851/55 | 10190/17 | 4653/9 |
|  |  | HR | **1.62** | 1.26 | 0.97 | 1 (ref) | 0.75 | 1.08 |
|  |  | 95% CI | (1.11-2.36) | (0.90-1.77) | (0.69-1.37) |  | (0.44-1.3) | (0.53-2.18) |
|  | Yes | N / n | 2378/20 | 5628/20 | 7955/44 | 5919/30 | 2946/10 | 1716/14 |
|  |  | HR | **2.00** | 0.84 | 1.14 | 1 (ref) | 0.66 | **2.10** |
|  |  | 95% CI | (1.12-3.55) | (0.47-1.47) | (0.72-1.82) |  | (0.32-1.35) | (1.11-3.96) |

All HRs were adjusted for age, behavior, income, and family history of cardiovascular disease. Ex-smoker group among women was not presented due to the small number. BMI, body mass index; HTN, hypertension; DM, diabetes mellitus; HR, hazard ratio.
