# Supplementary material for: Associations between body mass index and mortality or cardiovascular events in a general Korean population
Source: PLoS One. 2017 Sep 15;12(9):e0185024. doi: 10.1371/journal.pone.0185024 (PMC5600387; doi:10.1371/journal.pone.0185024)
Supplement: S7 Table — All HRs were adjusted for age, behavior, income, and family history of cardiovascular disease. Ex-smoker group among women was not presented due to the small number. BMI, body mass index; HTN, hypertension; DM, diabetes mellitus; HR, hazard ratio. (DOCX) [file pone.0185024.s007.docx]

Supplemental Table 7. Multivariate hazard ratios for the occurrence of a cardiovascular disease event according to body mass index

|  |  | BMI (kg/m^2^) | <20 | 20-22.4 | | | 22.5-24.9 | | 25-27.4 | | 27.5-29.9 | | | ≥30 | |
| --- | --- | --- | --- | --- | --- | --- | --- | --- | --- | --- | --- | --- | --- | --- | --- |
| **Men** |  |  |  |  | | |  | |  | |  | | |  | |
| All |  | N / n | 17725/1244 | 49352/2873 | | | 71313/4252 | | 53063/3416 | | 19371/1185 | | | 7564/423 | |
|  |  | HR | **1.08** | 1 (ref) | | | **1.07** | | **1.23** | | **1.34** | | | **1.67** | |
|  |  | (95% CI) | (1.01-1.16) |  | | | (1.02-1.12) | | (1.17-1.29) | | (1.25-1.43) | | | (1.51-1.85) | |
| Smoking | Non-smoker | N / n | 6115/405 | 19616/1100 | | | 31139/1860 | | 23822/1582 | | 8542/567 | | | 3091/186 | |
|  | (never, ex-) | HR | 1.06 | 1 (ref) | | | **1.08** | | **1.26** | | **1.41** | | | **1.68** | |
|  |  | (95% CI) | (0.94-1.18) |  | | | (1.00-1.16) | | (1.17-1.36) | | (1.27-1.56) | | | (1.44-1.96) | |
|  | Never smoker | N / n | 5017/359 | 15444/959 | | | 24020/1540 | | 18057/1316 | | 6458/474 | | | 2282/155 | |
|  |  | HR | 1.04 | 1 (ref) | | | 1.04 | | **1.25** | | **1.39** | | | **1.65** | |
|  |  | (95% CI) | (0.92-1.18) |  | | | (0.96-1.13) | | (1.15-1.36) | | (1.24-1.55) | | | (1.39-1.95) | |
|  | Ex-smoker | N / n | 1098/46 | 4172/141 | | | 7119/320 | | 5765/266 | | 2084/93 | | | 809/31 | |
|  |  | HR | 1.09 | 1 (ref) | | | **1.38** | | **1.45** | | **1.61** | | | **1.93** | |
|  |  | (95% CI) | (0.78-1.52) |  | | | (1.13-1.68) | | (1.18-1.78) | | (1.23-2.09) | | | (1.30-2.85) | |
|  | Current smoker | N / n | 10143/695 | 25228/1444 | | | 33116/1862 | | 23654/1429 | | 8936/478 | | | 3878/193 | |
|  |  | HR | 1.07 | 1 (ref) | | | 1.06 | | **1.24** | | **1.32** | | | **1.72** | |
|  |  | (95% CI) | (0.98-1.17) |  | | | (0.99-1.13) | | (1.16-1.34) | | (1.19-1.46) | | | (1.48-2.00) | |
| HTN | No | N / n | 14112/670 | 37663/1424 | | | 49964/1972 | | 33413/1406 | | 10788/416 | | | 3637/123 | |
|  |  | HR | **1.12** | 1 (ref) | | | **1.11** | | **1.27** | | **1.39** | | | **1.60** | |
|  |  | (95% CI) | (1.02-1.22) |  | | | (1.04-1.19) | | (1.18-1.37) | | (1.25-1.55) | | | (1.33-1.93) | |
|  | Yes | N / n | 3613/574 | 11689/1449 | | | 21349/2280 | | 19650/2010 | | 8583/769 | | | 3927/300 | |
|  |  | HR | **1.14** | 1 (ref) | | | 0.96 | | 1.04 | | 1.07 | | | **1.33** | |
|  |  | (95% CI) | (1.04-1.26) |  | | | (0.89-1.02) | | (0.97-1.12) | | (0.98-1.17) | | | (1.18-1.51) | |
| DM | No | N / n | 15796/966 | 43901/2167 | | | 62137/3213 | | 45250/2557 | | 16042/854 | | | 6055/280 | |
|  |  | HR | **1.11** | 1 (ref) | | | **1.10** | | **1.28** | | **1.38** | | | **1.62** | |
|  |  | (95% CI) | (1.02-1.19) |  | | | (1.04-1.16) | | (1.21-1.36) | | (1.28-1.50) | | | (1.43-1.84) | |
|  | Yes | N / n | 1929/278 | 5451/706 | | | 9176/1039 | | 7813/859 | | 3329/331 | | | 1509/143 | |
|  |  | HR | 1.08 | 1 (ref) | | | 0.93 | | 0.98 | | 1.04 | | | **1.42** | |
|  |  | (95% CI) | (0.94-1.24) |  | | | (0.84-1.02) | | (0.89-1.08) | | (0.91-1.19) | | | (1.19-1.70) | |
| **Women** |  |  |  | | |  | |  | |  | |  | |  |  |
| All |  | N / n | 30184/905 | | 58661/2337 | | 56999/3415 | | 31946/2483 | | 13210/1201 | | 6408/583 | | |
|  |  | HR | 0.93 | | 1 (ref) | | **1.15** | | **1.28** | | **1.49** | | **1.76** | | |
|  |  | (95% CI) | (0.86-1.00) | |  | | (1.09-1.21) | | (1.21-1.35) | | (1.39-1.6) | | (1.61-1.93) | | |
| Smoking | Non-smoker | N / n | 27598/781 | | 54213/2112 | | 52957/3119 | | 29676/2273 | | 12272/1101 | | 5850/544 | | |
|  | (never, ex-) | HR | **0.92** | | 1 (ref) | | **1.14** | | **1.27** | | **1.48** | | **1.81** | | |
|  |  | (95% CI) | (0.85-1.00) | |  | | (1.08-1.21) | | (1.2-1.35) | | (1.38-1.6) | | (1.65-1.99) | | |
|  | Never smoker | N / n | 27098/774 | | 53565/2108 | | 52416/3097 | | 29400/2261 | | 12141/1095 | | 5770/542 | | |
|  |  | HR | **0.92** | | 1 (ref) | | **1.14** | | **1.27** | | **1.48** | | **1.80** | | |
|  |  | (95% CI) | (0.84-0.99) | |  | | (1.08-1.20) | | (1.19-1.35) | | (1.37-1.59) | | (1.64-1.98) | | |
|  | Current smoker | N / n | 1539/80 | | 2333/120 | | 1861/143 | | 1066/103 | | 458/42 | | 318/19 | | |
|  |  | HR | 0.99 | | 1 (ref) | | **1.37** | | **1.42** | | **1.47** | | 1.30 | | |
|  |  | (95% CI) | (0.75-1.32) | |  | | (1.08-1.75) | | (1.09-1.85) | | (1.03-2.08) | | (0.80-2.10) | | |
| HTN | No | N / n | 26719/516 | | 48499/1280 | | 41262/1583 | | 19733/976 | | 7025/370 | | 2982/144 | | |
|  |  | HR | 0.92 | | 1 (ref) | | **1.15** | | **1.30** | | **1.47** | | **1.72** | | |
|  |  | (95% CI) | (0.83-1.01) | |  | | (1.07-1.24) | | (1.20-1.42) | | (1.31-1.65) | | (1.45-2.04) | | |
|  | Yes | N / n | 3465/389 | | 10162/1057 | | 15737/1832 | | 12213/1507 | | 6185/831 | | 3426/439 | | |
|  |  | HR | 1.03 | | 1 (ref) | | 1.07 | | **1.11** | | **1.26** | | **1.43** | | |
|  |  | (95% CI) | (0.91-1.15) | |  | | (0.99-1.16) | | (1.03-1.20) | | (1.15-1.38) | | (1.28-1.60) | | |
| DM | No | N / n | 27763/710 | | 52957/1842 | | 48957/2520 | | 25970/1733 | | 10241/797 | | 4676/355 | | |
|  |  | HR | **0.92** | | 1 (ref) | | **1.14** | | **1.26** | | **1.47** | | **1.72** | | |
|  |  | (95% CI) | (0.84-1.00) | |  | | (1.07-1.21) | | (1.18-1.34) | | (1.35-1.60) | | (1.53-1.93) | | |
|  | Yes | N / n | 2421/195 | | 5704/495 | | 8042/895 | | 5976/750 | | 2969/404 | | 1732/228 | | |
|  |  | HR | 1.02 | | 1 (ref) | | **1.12** | | **1.19** | | **1.33** | | **1.51** | | |
|  |  | (95% CI) | (0.86-1.20) | |  | | (1.00-1.25) | | (1.07-1.34) | | (1.17-1.52) | | (1.29-1.77) | | |

All HRs were adjusted for age, behavior, income, and family history of cardiovascular disease. Ex-smoker group among women was not presented due to the small number. BMI, body mass index; HTN, hypertension; DM, diabetes mellitus; HR, hazard ratio.
