# Supplementary material for: Associations between body mass index and mortality or cardiovascular events in a general Korean population
Source: PLoS One. 2017 Sep 15;12(9):e0185024. doi: 10.1371/journal.pone.0185024 (PMC5600387; doi:10.1371/journal.pone.0185024)
Supplement: S8 Table — All HRs were adjusted for age, behavior, income, and family history of cardiovascular disease. Ex-smoker group among women was not presented due to the small number. BMI, body mass index; HTN, hypertension; DM, diabetes mellitus; HR, hazard ratio. (DOCX) [file pone.0185024.s008.docx]

Supplemental Table 8. Multivariate hazard ratios for the occurrence of a cardiovascular disease event according to body mass index, excluding subjects who died or were diagnosed as the corresponding disease within less than 3 years after baseline examination

|  |  | BMI (kg/m^2^) | <20 | 20-22.4 | | 22.5-24.9 | | 25-27.4 | | | 27.5-29.9 | ≥30 |
| --- | --- | --- | --- | --- | --- | --- | --- | --- | --- | --- | --- | --- |
| **Men** |  |  |  |  | |  | |  | | |  |  |
| All |  | N / n | 16991/804 | 48006/1952 | | 69598/2917 | | 51830/2410 | | | 18910/799 | 7392/280 |
|  |  | HR | 1.06 | 1 (ref) | | **1.07** | | **1.26** | | | **1.33** | **1.69** |
|  |  | (95% CI) | (0.97-1.15) |  | | (1.01-1.13) | | (1.19-1.34) | | | (1.23-1.45) | (1.49-1.91) |
| Smoking | Non-smoker | N / n | 5866/264 | 19087/734 | | 30369/1267 | | 23239/1105 | | | 8314/372 | 3005/113 |
|  | (never, ex-) | HR | 1.05 | 1 (ref) | | **1.10** | | **1.33** | | | **1.41** | **1.61** |
|  |  | (95% CI) | (0.91-1.21) |  | | (1-1.2) | | (1.21-1.46) | | | (1.24-1.59) | (1.32-1.96) |
|  | Never smoker | N / n | 4800/236 | 15002/650 | | 23401/1074 | | 17591/932 | | | 6280/323 | 2211/95 |
|  |  | HR | 1.04 | 1 (ref) | | 1.06 | | **1.29** | | | **1.40** | **1.54** |
|  |  | (95% CI) | (0.89-1.2) |  | | (0.96-1.17) | | (1.17-1.43) | | | (1.22-1.6) | (1.24-1.91) |
|  | Ex-smoker | N / n | 1066/28 | 4085/84 | | 6968/193 | | 5648/173 | | | 2034/49 | 794/18 |
|  |  | HR | 1.14 | 1 (ref) | | **1.38** | | **1.58** | | | **1.46** | **2.04** |
|  |  | (95% CI) | (0.74-1.75) |  | | (1.07-1.79) | | (1.21-2.05) | | | (1.02-2.08) | (1.22-3.4) |
|  | Current smoker | N / n | 9738/446 | 24549/981 | | 32358/1260 | | 23140/1007 | | | 8747/323 | 3804/135 |
|  |  | HR | 1.05 | 1 (ref) | | 1.04 | | **1.26** | | | **1.30** | **1.82** |
|  |  | (95% CI) | (0.94-1.17) |  | | (0.95-1.13) | | (1.16-1.38) | | | (1.15-1.48) | (1.52-2.19) |
| HTN | No | N / n | 13716/437 | 37030/1006 | | 49188/1373 | | 32944/1043 | | | 10642/296 | 3592/90 |
|  |  | HR | 1.04 | 1 (ref) | | **1.09** | | **1.34** | | | **1.44** | **1.78** |
|  |  | (95% CI) | (0.93-1.16) |  | | (1.01-1.19) | | (1.23-1.46) | | | (1.27-1.64) | (1.43-2.21) |
|  | Yes | N / n | 3275/367 | 10976/946 | | 20410/1544 | | 18886/1367 | | | 8268/503 | 3800/190 |
|  |  | HR | **1.17** | 1 (ref) | | 0.97 | | 1.06 | | | 1.04 | **1.28** |
|  |  | (95% CI) | (1.03-1.32) |  | | (0.89-1.05) | | (0.97-1.15) | | | (0.93-1.16) | (1.09-1.5) |
| DM | No | N / n | 15267/647 | 42924/1501 | | 60899/2256 | | 44384/1852 | | | 15710/578 | 5941/191 |
|  |  | HR | **1.09** | 1 (ref) | | **1.10** | | **1.33** | | | **1.36** | **1.65** |
|  |  | (95% CI) | (0.99-1.2) |  | | (1.03-1.18) | | (1.24-1.42) | | | (1.23-1.5) | (1.42-1.92) |
|  | Yes | N / n | 1724/157 | 5082/451 | | 8699/661 | | 7446/558 | | | 3200/221 | 1451/89 |
|  |  | HR | 1.01 | 1 (ref) | | 0.90 | | 0.96 | | | 1.04 | **1.36** |
|  |  | (95% CI) | (0.84-1.21) |  | | (0.79-1.01) | | (0.85-1.09) | | | (0.88-1.22) | (1.08-1.7) |
| **Women** |  |  |  | |  | |  | |  |  | |  |
| All |  | N / n | 29737/581 | 57672/1548 | | 55748/2328 | | 31026/1685 | | | 12746/782 | 6177/374 |
|  |  | HR | 0.92 | 1 (ref) | | **1.17** | | **1.29** | | | **1.46** | **1.75** |
|  |  | (95% CI) | (0.84-1.01) |  | | (1.1-1.25) | | (1.2-1.38) | | | (1.34-1.6) | (1.56-1.96) |
| Smoking | Non-smoker | N / n | 27219/502 | 53320/1395 | | 51819/2127 | | 28826/1535 | | | 11838/709 | 5634/349 |
|  | (never, ex-) | HR | 0.92 | 1 (ref) | | **1.17** | | **1.29** | | | **1.44** | **1.79** |
|  |  | (95% CI) | (0.83-1.02) |  | | (1.09-1.25) | | (1.19-1.38) | | | (1.32-1.58) | (1.59-2.02) |
|  | Never smoker | N / n | 26727/501 | 52677/1393 | | 51289/2115 | | 28555/1527 | | | 11711/707 | 5554/347 |
|  |  | HR | 0.92 | 1 (ref) | | **1.16** | | **1.28** | | | **1.44** | **1.78** |
|  |  | (95% CI) | (0.83-1.02) |  | | (1.09-1.25) | | (1.19-1.38) | | | (1.32-1.58) | (1.59-2.01) |
|  | Current smoker | N / n | 1498/53 | 2272/79 | | 1804/100 | | 1024/67 | | | 441/28 | 311/12 |
|  |  | HR | 1.04 | 1 (ref) | | **1.44** | | 1.33 | | | 1.36 | 1.20 |
|  |  | (95% CI) | (0.73-1.48) |  | | (1.07-1.93) | | (0.96-1.84) | | | (0.88-2.1) | (0.65-2.22) |
| HTN | No | N / n | 26476/332 | 48006/886 | | 40758/1151 | | 19414/704 | | | 6877/239 | 2935/102 |
|  |  | HR | **0.86** | 1 (ref) | | **1.18** | | **1.31** | | | **1.33** | **1.79** |
|  |  | (95% CI) | (0.76-0.98) |  | | (1.08-1.29) | | (1.18-1.44) | | | (1.15-1.54) | (1.46-2.2) |
|  | Yes | N / n | 3261/249 | 9666/662 | | 14990/1177 | | 11612/981 | | | 5869/543 | 3242/272 |
|  |  | HR | 1.08 | 1 (ref) | | **1.09** | | **1.15** | | | **1.32** | **1.44** |
|  |  | (95% CI) | (0.94-1.26) |  | | (0.99-1.2) | | (1.04-1.27) | | | (1.17-1.47) | (1.25-1.66) |
| DM | No | N / n | 27427/463 | 52218/1253 | | 48095/1761 | | 25373/1220 | | | 9949/535 | 4543/237 |
|  |  | HR | **0.89** | 1 (ref) | | **1.15** | | **1.27** | | | **1.43** | **1.7** |
|  |  | (95% CI) | (0.8-1) |  | | (1.07-1.24) | | (1.17-1.37) | | | (1.29-1.58) | (1.48-1.95) |
|  | Yes | N / n | 2310/118 | 5454/295 | | 7653/567 | | 5653/465 | | | 2797/247 | 1634/137 |
|  |  | HR | 1.09 | 1 (ref) | | **1.19** | | **1.23** | | | **1.36** | **1.54** |
|  |  | (95% CI) | (0.88-1.35) |  | | (1.03-1.37) | | (1.06-1.43) | | | (1.15-1.61) | (1.25-1.88) |

All HRs were adjusted for age, behavior, income, and family history of cardiovascular disease. Ex-smoker group among women was not presented due to the small number. BMI, body mass index; HTN, hypertension; DM, diabetes mellitus; HR, hazard ratio.
