# Supplementary material for: Associations between body mass index and mortality or cardiovascular events in a general Korean population
Source: PLoS One. 2017 Sep 15;12(9):e0185024. doi: 10.1371/journal.pone.0185024 (PMC5600387; doi:10.1371/journal.pone.0185024)
Supplement: S1 Fig — HRs were adjusted for gender, health behaviors (smoking, alcohol consumption, physical activity), income, and family history of CVD. BMI, body mass index; CVD, cardiovascular disease; DM, diabetes mellitus; HR, hazard ratio; HTN, hypertension. (DOCX) [file pone.0185024.s009.docx]

Supplement figure 1. Overall mortality risk according to smoking status, hypertension, and diabetes mellitus status


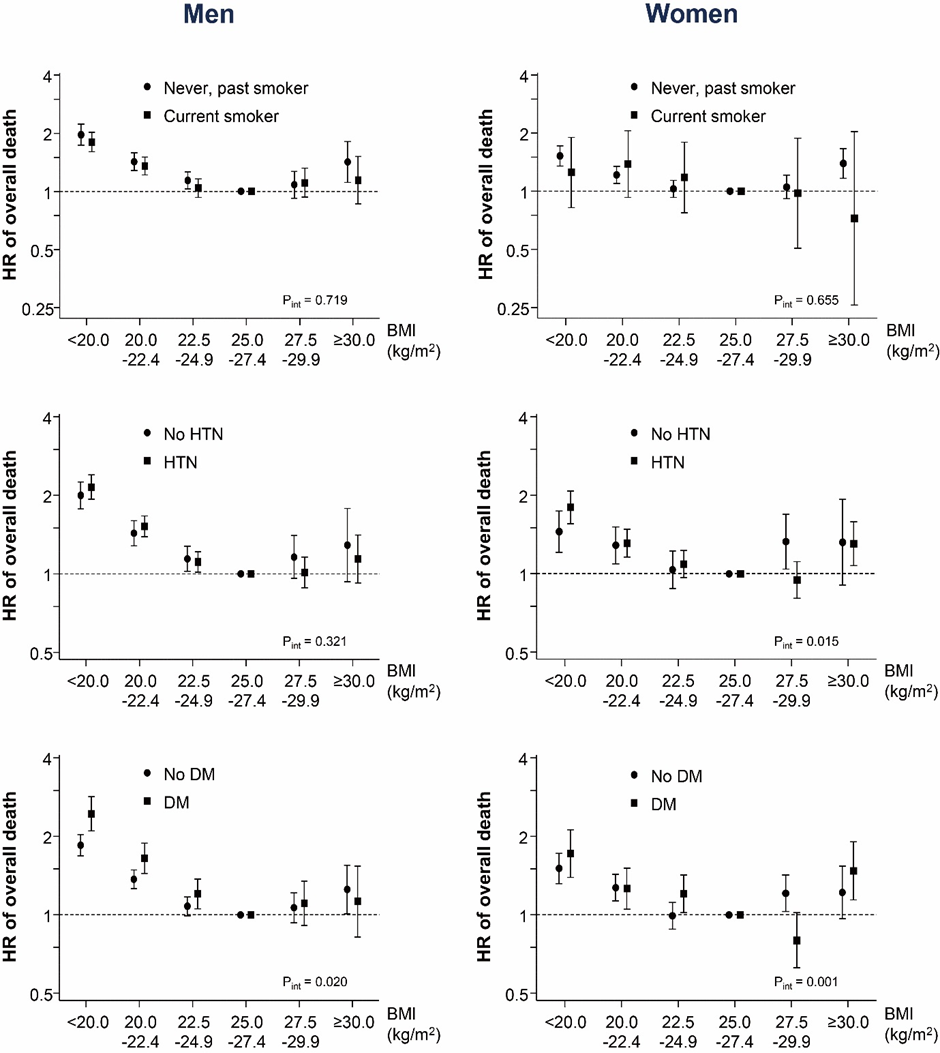


HRs were adjusted for gender, health behaviors (smoking, alcohol consumption, physical activity), income, and family history of CVD.

BMI, body mass index; CVD, cardiovascular disease; DM, diabetes mellitus; HR, hazard ratio; HTN, hypertension
